# Alterations in Sub-Axonal Architecture Between Normal Aging and Parkinson’s Diseased Human Brains Using Label-Free Cryogenic X-ray Nanotomography

**DOI:** 10.3389/fnins.2020.570019

**Published:** 2020-11-25

**Authors:** Hung Tri Tran, Esther H. R. Tsai, Amanda J. Lewis, Tim Moors, J. G. J. M. Bol, Iman Rostami, Ana Diaz, Allert J. Jonker, Manuel Guizar-Sicairos, Joerg Raabe, Henning Stahlberg, Wilma D. J. van de Berg, Mirko Holler, Sarah H. Shahmoradian

**Affiliations:** ^1^Paul Scherrer Institut, Villigen, Switzerland; ^2^Center for Cellular Imaging and NanoAnalytics (C-CINA), Biozentrum, University of Basel, Basel, Switzerland; ^3^Department of Anatomy and Neurosciences, Section Clinical Neuroanatomy, Amsterdam Neuroscience, Amsterdam UMC, Vrije Universiteit Amsterdam, Amsterdam, Netherlands

**Keywords:** human brain, neurodegeneration, Tomography – X-ray computed, Parkinson’s and related diseases, label-free, electron microscograpy, ptychography, axons

## Abstract

Gaining insight to pathologically relevant processes in continuous volumes of unstained brain tissue is important for a better understanding of neurological diseases. Many pathological processes in neurodegenerative disorders affect myelinated axons, which are a critical part of the neuronal circuitry. Cryo ptychographic X-ray computed tomography in the multi-keV energy range is an emerging technology providing phase contrast at high sensitivity, allowing label-free and non-destructive three dimensional imaging of large continuous volumes of tissue, currently spanning up to 400,000 μm^3^. This aspect makes the technique especially attractive for imaging complex biological material, especially neuronal tissues, in combination with downstream optical or electron microscopy techniques. A further advantage is that dehydration, additional contrast staining, and destructive sectioning/milling are not required for imaging. We have developed a pipeline for cryo ptychographic X-ray tomography of relatively large, hydrated and unstained biological tissue volumes beyond what is typical for the X-ray imaging, using human brain tissue and combining the technique with complementary methods. We present four imaged volumes of a Parkinson’s diseased human brain and five volumes from a non-diseased control human brain using cryo ptychographic X-ray tomography. In both cases, we distinguish neuromelanin-containing neurons, lipid and melanic pigment, blood vessels and red blood cells, and nuclei of other brain cells. In the diseased sample, we observed several swellings containing dense granular material resembling clustered vesicles between the myelin sheaths arising from the cytoplasm of the parent oligodendrocyte, rather than the axoplasm. We further investigated the pathological relevance of such swollen axons in adjacent tissue sections by immunofluorescence microscopy for phosphorylated alpha-synuclein combined with multispectral imaging. Since cryo ptychographic X-ray tomography is non-destructive, the large dataset volumes were used to guide further investigation of such swollen axons by correlative electron microscopy and immunogold labeling post X-ray imaging, a possibility demonstrated for the first time. Interestingly, we find that protein antigenicity and ultrastructure of the tissue are preserved after the X-ray measurement. As many pathological processes in neurodegeneration affect myelinated axons, our work sets an unprecedented foundation for studies addressing axonal integrity and disease-related changes in unstained brain tissues.

## Introduction

Multi-scale visualization of the hierarchical organization of human brain is critical to neuroscience. Micro- and nanomorphology of the neuronal network is tightly linked with the brain’s functionality, from the macroscopic level, i.e., specialized populations of neurons, to the nanoscopic level, i.e., synaptic connections between individual neurons. Large-scale, label-free 3D imaging at nanoscale resolution of near-native state tissues can reveal new insights to such hierarchically organized neuronal structures.

The neuronal network of the human brain is complex. Neurons communicate via their extensions known as dendrites or axons. Axons are often wrapped in segments of lipid membrane sheaths known as myelin, which provide it with insulating and stabilizing properties. Myelin sheaths are essentially flattened portions of extensions of the cell membrane of oligodendrocytes. The high lipid content of myelin sheaths encasing the axon serves to enhance conduction velocity (Nave and Werner, 2014). Myelinated axons are a critical part of the neuronal circuitry and constitute approximately 40% of the human brain (Morell and Norton, 1980).

Neurites, especially myelinated axons, are a critical functional component of brain cells, serving as highways of cross-communication. They relay physical cargo and electrical signals from one neuron to another. Pathologically related aggregation processes within the axon can cause a localized swelling that interferes with normal trafficking (Chevalier-Larsen and Holzbaur, 2006). Alzheimer’s disease, Parkinson’s disease, and multiple sclerosis are a few examples of such diseases (Su et al., 1993; Adalbert et al., 2009; Inglese and Petracca, 2013; Lee et al., 2014; Friese, 2016) in which axonal integrity is affected. The ability to image several axons and their inner contents at once is particularly advantageous when applied to diseased and degenerative brain conditions in which neurites are pathologically involved or affected. Capturing a wide and inclusive view of features in normal and dystrophic axons within the tissue can permit new insights to the axonal component of pathology in neurodegenerative diseases, which is not well understood.

Synchrotron X-ray micro-tomography is an imaging technique that can be used to map neural circuits in brain tissue with reported resolutions of down to 1 μm, using heavy-metal staining such as silver nitrate for contrast enhancement and oftentimes plastic- or paraffin-embedding for rigid preservation (Mizutani et al., 2009; de Castro Fonseca et al., 2018). Phase contrast has also been used in propagation-based X-ray imaging to obtain high-quality 3D images of myelinated axons (Dyer et al., 2017). However, the resolution in these techniques is insufficient for a detailed morphological characterization of axons. Lens-based X-ray microscopy makes use of X-ray optics for magnification, as in holo-tomography, for example (Khimchenko et al., 2018). At the water window, i.e., at photon energies between 281 and 533 eV, carbon-rich structures in biological tissue exhibit a high contrast compared to water. In this way, biological matter can be imaged in 3D at a resolution of about 30 nm, albeit with a depth limited to a couple of micrometers (Le Gros et al., 2016; Pérez-Berná et al., 2016).

However, for imaging myelinated axons, volumes of several tens of micrometers are required while preserving a high spatial resolution, for which harder X-rays with photon energies above about 2 keV are necessary. Despite the difficulty to fabricate optimally efficient lenses for hard X-rays, lens-based microscopy of neural tissue has been demonstrated (Wu et al., 2012). Additionally, propagation-based hard X-ray microscopy can be achieved by using a divergent beam to produce magnified images (Mokso et al., 2007; Bartels et al., 2015), which allowed resolving myelinated axons within resin-embedded nerves specimens (Bartels et al., 2015; Kuan et al., 2020).

In contrast, ptychographic X-ray computed tomography (PXCT) is a lens-less technique in which spatial resolution is not limited by imaging optics. It currently allows reaching a resolution down to about 15 nm in 3D (Holler et al., 2014, 2017a) on specimens that exhibit small features with sufficient density contrast. As density contrast is small in biological samples for hard X-rays, resolution has been typically limited to the 100 nm range in both stained, resin-embedded and frozen-hydrated specimens (Diaz et al., 2015; Pfister et al., 2016; Shahmoradian et al., 2017b). However, the sensitivity of PXCT is high enough to visualize ultrastructural features in fully hydrated samples without requiring heavy metal staining for contrast purposes, or destruction of material (ion beam milling, sectioning) for accessing tissue depths (Shahmoradian et al., 2017b). These are important factors for enabling multi-scale downstream processing in a relatively minimally perturbed state. The OMNY instrument (Holler et al., 2018) allows cryogenic PXCT (cryo-PXCT) measurements of biological samples in cryo conditions and under vacuum, forming a powerful label-free microscopy technique.

All of the resulting ultrastructural information from cryo-PXCT data can be correlated to electron density, directly interpretable from the grayscale values of the tomographic data, which can be, in turn, related to its local mass density using reasonable assumptions about the stoichiometric composition (Diaz et al., 2015). This is useful for attributing an identity to each ultrastructural feature within the complex and crowded tissue landscape. Cryo-PXCT at photon energies between about 6 and 8 keV has the ability to provide information across relatively large volumes of unstained tissue, enabling the imaging of multiple cell bodies and the tracking of morphological intracellular changes along the lengths of radiating structures such as cellular extensions; in the case of brain tissue, along the length of neuronal processes.

Using cryo-PXCT, the visualization of tissue contents including myelinated axons at a resolution in the 100 nm range is independent of pre-marking selected features using pigment-based or fluorescent labels (Kremers et al., 2011; Shu et al., 2011; Manning et al., 2012; Lam et al., 2015). This enables a wide and inclusive view of numerous features existing in the tissue, including normal and dystrophic axons, which are otherwise easily missed or unintentionally excluded.

Parkinson’s disease is a complex neurodegenerative disease, the second most common after Alzheimer’s disease, in which the axonal component of pathology is not well understood. While axonal pathology, including swollen (dystrophic) axons and alterations in axonal transport, have been extensively noted in Parkinson’s disease patients, animal and cell culture-based models, little is known beyond the facts that they can appear physically swollen, have slower vesicular transport and contain aggregated material including alpha-synuclein (aSyn), beta-synuclein, and gamma-synuclein (Galvin et al., 1999; Kotzbauer et al., 2004; Saha et al., 2004; Chung et al., 2009; Burke and O’Malley, 2013; Koch et al., 2015; Sekigawa et al., 2015; Tagliaferro and Burke, 2016; Kouroupi et al., 2017). Correlative light and electron microscopy of Parkinson’s diseased human brain tissue sections has recently shown such dystrophic axons, specifically Lewy neurites, to contain vesicular structures, dysmorphic mitochondria and disrupted cytoskeletal elements (Shahmoradian et al., 2017a). However, imaging several dystrophic axons and their contained ultrastructures simultaneously is not efficient by electron microscopy alone. Cryo-PXCT enables the simultaneous visualization of several axons and the subtleties within and throughout each axon that are easily missed unless visualizing a continuous length, i.e., across tens of microns, up to (100 μm)^3^. Such subtleties include, but are not limited to, disruptions or abnormalities within the myelin sheath wrappings at specific points along the length of the axon, or abnormalities within the axon itself.

Beyond generating a 3D picture of multiple axons and brain cells to recognize pathologically relevant features, cryo-PXCT allows for the tissue to remain intact at the nanoscale after imaging. This aspect opens up the unique possibility of downstream processing of selected features of interest by higher resolution techniques such as electron microscopy, as also shown herein, or gaining biochemical information by different spectroscopic-based imaging approaches, such as Fourier-transform infrared spectroscopy (FTIR), coherent anti-Stokes Raman spectroscopy (CARS), and matrix-assisted laser desorption/ionization mass spectroscopy imaging (MALDI-IMS).

Starting with several chemically fixed, hydrated tissue blocks from a postmortem Parkinson’s diseased human brain and from a control/non-demented human brain, we processed and imaged these by cryo-PXCT, followed by cryo-ultramicrotomy and cryo-immunogold labeling and electron microscopy. We thus demonstrate a multi-scale imaging pipeline using cryo-PXCT followed by immuno-electron microscopy, with a cross investigation of pathological features of interest using fluorescence microscopy combined with multispectral imaging.

## Results

### Cryo-PXCT Imaging, Feature Segmentation and Quantification

**FIGURE 1:**
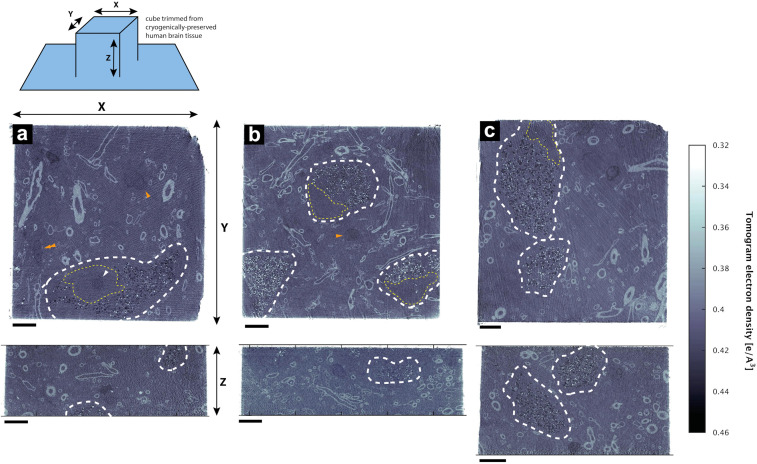
Hydrated, unstained non-demented human brain as imaged by cryo-PXCT using OMNY (Holler et al., 2018). Trimmed tissue volume schematic corresponding to orthoslice views is shown above. Representative orthoslices are shown from an X-Y plane (top panels) and X-Z plane (bottom panels) from three of the nine total human brain samples measured. Grayscale intensity values directly correspond to electron density. Data shown corresponds to orthoslices from **(a)** Tomo 5, **(b)** Tomo 1, **(c)** Tomo 4 (Table 1). Scale bars: 10 μm.

Cryo-PXCT using the OMNY (tOMography Nano crYo) instrument (Holler et al., 2018) at the cSAXS beamline of the Swiss Light Source was utilized to visualize five and four brain tissue samples, respectively (Table 1) from a control, non-demented donor and a Parkinson’s diseased donor (Table 2, Donors B and D). Cryo-PXCT was used to identify pathological-related abnormalities in postmortem brain tissue from Parkinson’s diseased (PD) human patients, within roughly cubic volumes spanning ∼(100 μm)^3^ at a resolution ranging from 145 to 390 nm. The *substantia nigra pars compacta* (SNpc) brain region was selected for dissection and imaging since this region typically contains the most Lewy pathology in the context of dopaminergic degeneration (Fearnley and Lees, 1991). Samples were prepared according to a pre-established protocol optimal for cryo-PXCT imaging of mouse brain tissue (Shahmoradian et al., 2017b), with an improved trimming procedure (Figure 1) for more efficient imaging.

**TABLE 1:**
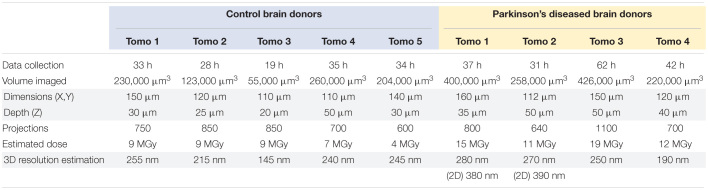
Imaging parameters and characteristics of biological samples imaged by cryo-PXCT using OMNY.

*Table represents the volume imaged per tomogram, data collection time, dimensions in X-Y-Z of the imaged region of the tissue block, projections used for tomographic reconstruction, estimated dose (MGy), and estimated 3D resolution, for each of the samples. Tomo 5 (control human brain, Movie 1) and Tomo 4 (Parkinson’s diseased human brain, Movie 5), shown in black-outlined rectangles, were selected for 3D color segmentation. Control: Tomo 1 = Movie 2; Tomo 2 = Movie 3; Tomo 3 = movie not shown; Tomo 4 = Movie 4; Tomo 5 = Movie 1. Parkinson’s diseased: Tomo 1 = Movie 6; Tomo 2 = Movie 7; Tomo 3 = Movie 8.*

**TABLE 2:** Clinical and pathological characteristics of brain donors.

Donor	Diagnosis	Age at onset (years)	Age at death (years)	Sex	PMD (hrs:min)	Braak aSyn stage
*A*	PDD	59	77	M	5:15	6
*B*	PDD	75	90	F	4:45	6
*C*	NDC	–	85	F	6:25	0
*D*	NDC	–	92	M	7:45	0
*E*	NDC	–	89	F	13:00	0

*PDD, Parkinson’s disease with dementia; age-at-onset, age at clinical diagnosis of PD; n.a., not applicable; NDC, non-demented control; aSyn, α-synuclein; PMD, postmortem delay. Age at onset, age at clinical diagnosis of PD. Donors B and D were used for cryo-PXCT and cryo immuno-electron microscopy. All donors were used for optical microscopy studies.*

Four cryo-PXCT tomographic datasets were generated from four separate blocks of human postmortem brain tissue from a PD brain donor (Table 1, Movies 5–8). As a control, five cryo-PXCT tomographic datasets were generated from five separate blocks of human postmortem brain tissue from a non-demented, age-matched control human brain donor (Table 1, Movies 1–4), three of which are shown as virtual slices in Figure 1.

**FIGURE 2:**
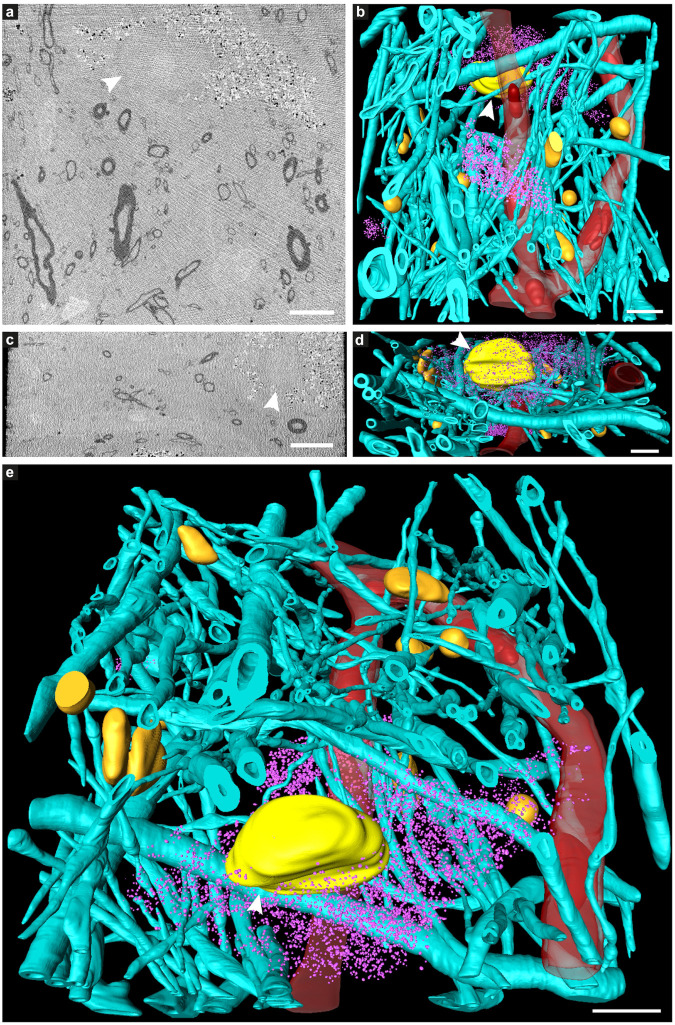
Tissue components in 3D color-segmented cryo-PXCT datasets of non-demented control human brain. Single 2D orthoslices with inverted grayscale in the **(a)** X-Y plane and **(c)** X-Z plane, shown from the 3D volume used for the corresponding color segmentation, displayed in the **(b)** X-Y plane, **(d)** X-Z plane, and **(e)** tilted larger-scale view. Aqua = myelinated axons; Yellow (white arrowhead) = nucleus of neuromelanin-containing cell; Orange = nuclei of non-neuromelanin- containing cells; Pink = neuromelanin-containing organelles; Red = blood vessels; Dark red = blood cells within the blood vessels. Corresponds to “Control human brain,” Tomo 5 (Table 1). Scale bars: 10 μm.

**FIGURE 3:**
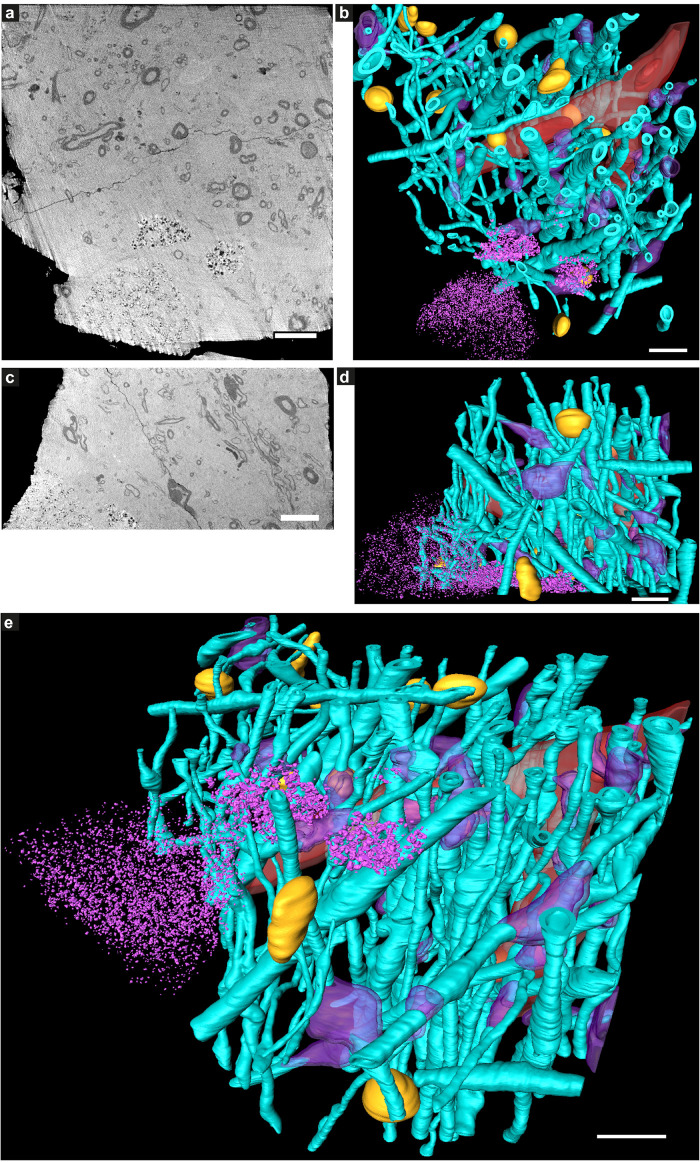
Tissue components in 3D color-segmented cryo-PXCT tomograms of Parkinson’s diseased human brain. Single 2D orthoslices with inverted grayscale in the **(a)** X-Y plane and **(c)** X-Z plane, shown from the 3D volume used for the corresponding color segmentation, displayed in the **(b)** X-Y plane, **(d)** X-Z plane, and **(e)** tilted larger-scale view. Aqua = myelinated axons; Purple = swellings along the axons (DMAs); Orange = nuclei of non-neuromelanin-containing cells; Pink = neuromelanin-containing organelles; Red = blood vessels; Dark red = blood cells within the blood vessels. Corresponds to “Parkinson’s diseased human brain,” Tomo 4 (Table 1). Grayscale shown herein does not correspond directly to mass density as opposed to Figure 1. Scale bars: 10 μm.

The tomograms showed neuromelanin-containing cells typical of the *substantia nigra* region (Figure 1, white dotted lines), as well as the cross-sections of myelinated axons (high contrast ellipsoid and circular structures). Nuclei (Figure 1, yellow dotted lines) of these neuromelanin-containing cells, and a smaller dense nucleolus (Figure 1a, white arrowhead), were also visible. Nuclei likely corresponding to microglial cells were observed as an elongated, irregular nucleus with characteristic peripheral heterochromatin and heterochromatin net made of multiple fused granules (García-Cabezas et al., 2016) (Figure 1, orange double-arrowhead), and dense, round nuclei were also visible (Figure 1, orange single-arrowheads). Following 3D color segmentation of the tomograms, the identity of several other features became more apparent. One tomogram from each group (control and PD) was selected for subsequent 3D color segmentation (Figures 2, 3, Movies 1 and 5, respectively).

**FIGURE 4:**
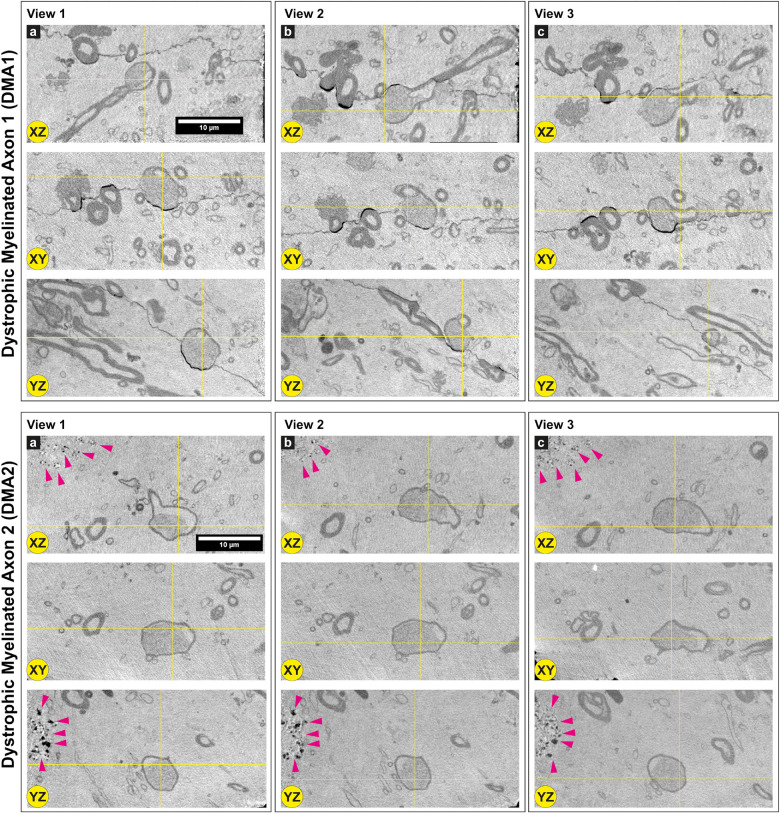
Dystrophic myelinated axons (DMAs) by cryo-PXCT in Parkinson’s diseased human brain. Single 2D orthoslices with inverted grayscale showing the appearance of two DMAs in different planes (X-Z, X-Y, Y-Z) and different positions (Views 1, 2, 3) in the 3D tomogram. Yellow crosses indicate the position of the DMA in the different views and planes. Several other DMAs are shown in Supplementary Figures 1, 2. Grayscale of all 2D cutaways shown herein correspond to electron density. Pink arrowheads = neuromelanin-containing organelles of adjacent cell. Scale bars: 10 μm. a, b, c refer to the different columns corresponding to View 1, View 2, and View 3, for clarity sake.

**FIGURE 5:**
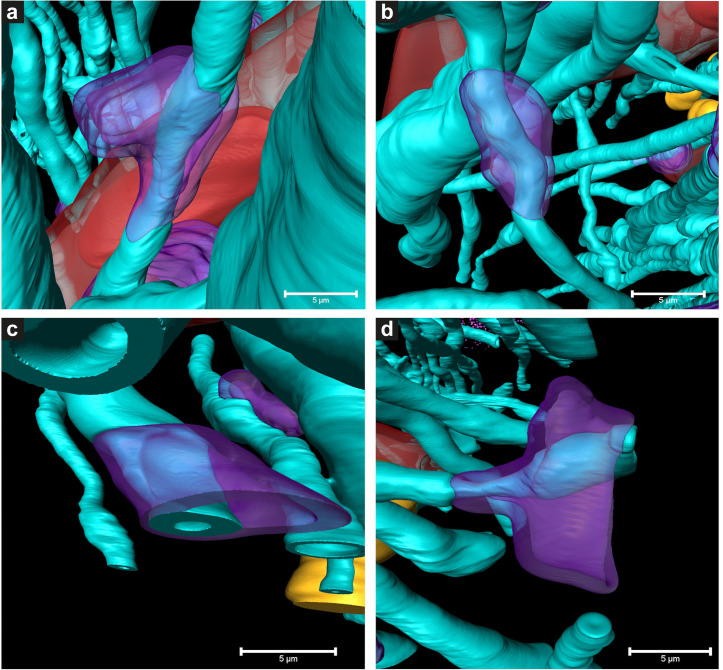
3D color representations of DMAs in Parkinson’s diseased human brain by cryo-PXCT. **(a–c)** DMAs in which the oligodendrocyte cytoplasm within the myelin sheaths is swollen (purple) at a region along the length of the myelinated axon (aqua), **(d)** DMA in which swelling is visible in both the oligodendrocyte cytoplasm (purple) and underlying axoplasm (aqua swelling beneath the purple). Red = blood vessel; Dark red = blood cell; Orange = nuclei of non-neuromelanin containing cells. Scale bars = 5 μm.

While features such as neuromelanin-containing cells, blood vessels, glial nuclei, and myelinated axons could all similarly be segmented from both control and PD cryo-PXCT human brain tomograms (Figures 2, 3), one feature that was uniquely observed in all four PD brain datasets as compared to all five control brain datasets was the presence of swellings within the myelinated axons (Figures 3b,d, purple segments within the aqua axons; Figure 4 and Supplementary Figures 1, 2, yellow crosses) as compared to the rest of the axons (Figures 3b,d, aqua axons). These swellings were in close proximity to the neuromelanin-containing cells, which are majorly affected in PD. Different views of approximately ten of these swellings, or dystrophic myelinated axons (DMAs) are shown at greater detail within one of the PD brain tomograms (Figure 4 and Supplementary Figures 1, 2; yellow crosses). The variability of the type of swelling is more apparent when the DMAs are visualized in 3D color segmentations, four of which are shown in Figure 5. For example, the inner part of the axon, or axoplasm, can appear relatively “normal” and consistent in diameter along the length of the axon (Figures 5a–c) with the swelling rather occurring in the layers comprising the myelin sheath of the axon, or the axoplasm itself can also become swollen (Figure 5d).

**FIGURE 6:**
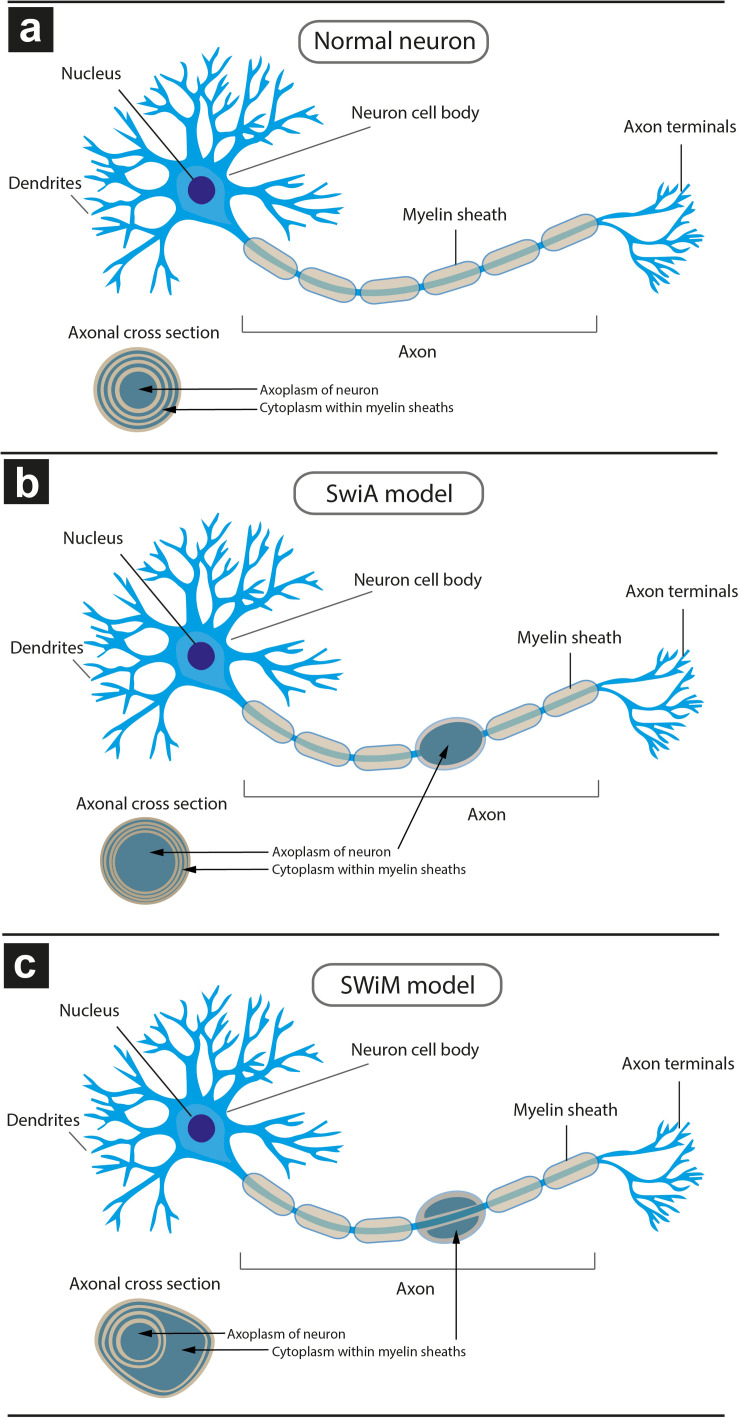
Schematic model of different types of dystrophic myelinated axons (DMAs) as observed by cryo-PXCT in Parkinson’s diseased human brain. Overall appearance of the myelinated axon protruding from a neuron, and zoomed-in cross-section of the myelinated axon (inset), is shown for each type: **(a)** neuron with a normal appearance of myelinated axon, in which the cytoplasm of the oligodendroglial cell is barely visible due to the typical tight compaction of the myelin sheaths (beige); **(b)** neuron with swelling in the axon, in which the neuronal cytoplasm within the axon is enlarged, referred to as “swelling in axoplasm” or SWiA; **(c)** neuron in which cytoplasm within myelin sheath is expanded, referred to as “swelling in myelin sheath” or SWiM. Oligodendroglial cells are not shown. Their extensions flatten out and wrap around the axon multiple times, forming the myelinations that appear as “sausage”-like pieces (beige) from the overview. The layers of myelin are apparent from the cross-sectional inset views.

Two types of DMAs were identified and characterized by cryo-PXCT: one population we refer to as SWiA (swollen in axoplasm) and another as SWiM (swollen in myelin). “In axoplasm” refers to cytoskeletal part of the axon that is in direct continuation from the neuron (Figure 6b), whereas “in myelin cytoplasm” refers to the cytoplasm within the myelin sheaths (Figure 6c) corresponding to that of the parent oligodendrocyte rather than the neuron (Fields, 2014). Since the resolution of these reconstructed tomograms enables distinguishing the axoplasm from the myelin sheath, especially when the myelin sheath appears to have partially separated, we could detect dense granular cytoplasmic material comprising these DMAs that appeared surprisingly often within the wrappings of the myelin sheath (Figures 4a–c and Supplementary Figure 1 all excluding A–C, 2) of the parent oligodendrocyte, hence the SWiM type, rather than simply within the axonal passage itself (the SWiA type). This was counter-intuitive considering that the clogging of the axon was not mainly occurring in the axonal passage itself, but rather in the exterior myelin sheaths compromising the axon.

Typically, axonal traffic jams associated with neurodegenerative disease are generally attributed to material accumulating within the axonal passage itself (Kuusisto et al., 2003; Chevalier-Larsen and Holzbaur, 2006; Doorn et al., 2014) thereby resulting in a local swelling of the SWiA type, which we did observe albeit to a lesser extent (Figure 4 “DMA2” A–C and Supplementary Figures 1A–C). Overall, 74% (40) of DMAs were found to be the SWiM type (Figure 6c), corresponding to a swelling within the cytoplasm of the actual myelin sheath, while 26% (14) of DMAs were found to correspond to the SWiA type (Figure 6b), corresponding to the more intuitive case of a swelling within the neuronal cytoplasm inside the axon itself (Supplementary Figure 4A). Quantification performed for each of the four PD brain tomograms independently (Supplementary Figures 4B–E) also showed a similar theme with the majority of DMAs corresponding to the SWiM rather than the SWiA type. To assess the degree of swelling in each DMA, three groups of measurements were taken for each DMA before, within, and after the swelling: (1) the axon diameter, averaged across three positions on the axon preceding the swelling, (2) of the maximum diameter of the axonal swelling, and (3) the axon diameter, averaged across three positions on the axon following the swelling. A great variability was observed for each swelling, ranging from ∼3 to 11 μm (Supplementary Figure 4).

### Optical Imaging: Alpha Synuclein Immunofluorescence With Bielschowsky Silver Staining

**FIGURE 7:**
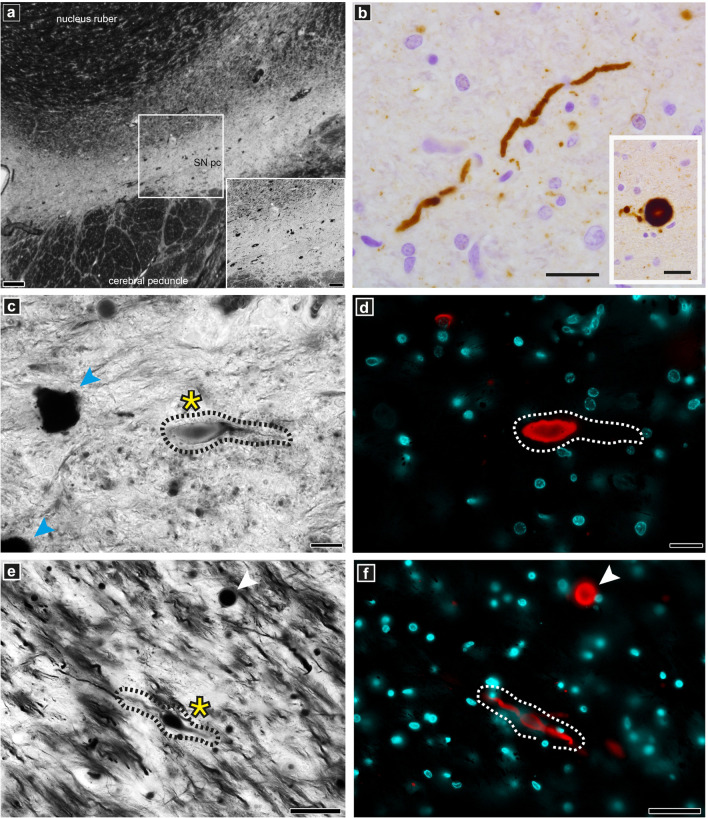
Optical microscopy and immunofluorescence of pathologically relevant structures in PD brain donor tissue. Staining and imaging was performed on adjacent tissue sections from the same PD brain donor SNpc region as used for the cryo-PXCT and EM studies. **(a)** overview of Bielschowsky silver staining of the SNpc. **(b)** Lewy neurite and Lewy body (insert) as revealed by p-aSyn immunohistochemistry. **(c,e)** Representative example of a dystrophic axon (black dotted lines, yellow asterix) visible by silver staining corresponding to be **(d,f)** Lewy neurite (white dotted lines) by fluorescence microscopy with spectral imaging. Lewy body is visible (white arrowhead) in both panels **(e)** and **(f)**. Neuromelanin-containing cells (blue arrowheads) are visible in panel **(c)**. Scale bars: **(a)** = 200 μm, inset 100 μm; **(b)** = 25 μm; **(c,d)** = 20 μm; **(e,f)** = 50 μm.

The DMAs were only observed in the PD human brain cryo-PXCT tomograms and were not present in the control human brain tomograms. To further investigate the pathological relevance of DMAs, we applied immunohistochemistry using a phosphorylated alpha-synuclein antibody (p-aSyn, Ser129-P aSyn, 11A5, gift from Prothena), together with a Bielschowsky silver staining protocol (Uchihara, 2007) on adjacent sections from same tissues as used for the X-ray/EM studies, in order to co-visualize nerve fibers as well as p-aSyn-immunopositive Lewy neurites in adjacent tissue blocks to those collected for cryo-PXCT. Stainings were analyzed by fluorescent microscopy – in combination with multispectral imaging – and confocal microscopy. The Bielschowsky staining showed the thickness and direction of axons originating from and directed to the SNpc. In a subset of p-aSyn immunopositive Lewy neurites, immunoreactivity overlapped partially or completely with swollen axons revealed by Bielschowsky stain, demonstrating the presence of p-aSyn inclusions in these dysmorphic processes (Figure 7).

### Correlative Electron Microscopy

For imaging downstream target structures at higher resolution, one PD and one control tissue block after cryo-PXCT imaging were selected for subsequent cryo ultramicrotomy at −100°C (Tokuyasu, 1980). The blocks were transferred and thawed at room temperature as routine for cryo-immunogold electron microscopy (Peters et al., 2006). Structures of interest were labeled by primary antibody followed by secondary immunogolds and contrast-stained by uranyl acetate (Peters and Pierson, 2008), then imaged by transmission electron microscopy (TEM) at ambient temperature. This workflow is shown in Supplementary Figure 5. For imaging downstream target structures at higher resolution, one PD and one control tissue block after cryo-PXCT imaging were selected for subsequent cryo ultramicrotomy at −100°C (Tokuyasu, 1980), transferred and thawed at room temperature as routine for cryo-immunogold electron microscopy (Peters et al., 2006), labeled by primary antibody followed by secondary immunogolds and contrast-stained by uranyl acetate (Peters and Pierson, 2008), then imaged by transmission electron microscopy (TEM) at ambient temperature. This workflow is shown in Supplementary Figure 5.

**FIGURE 8:**
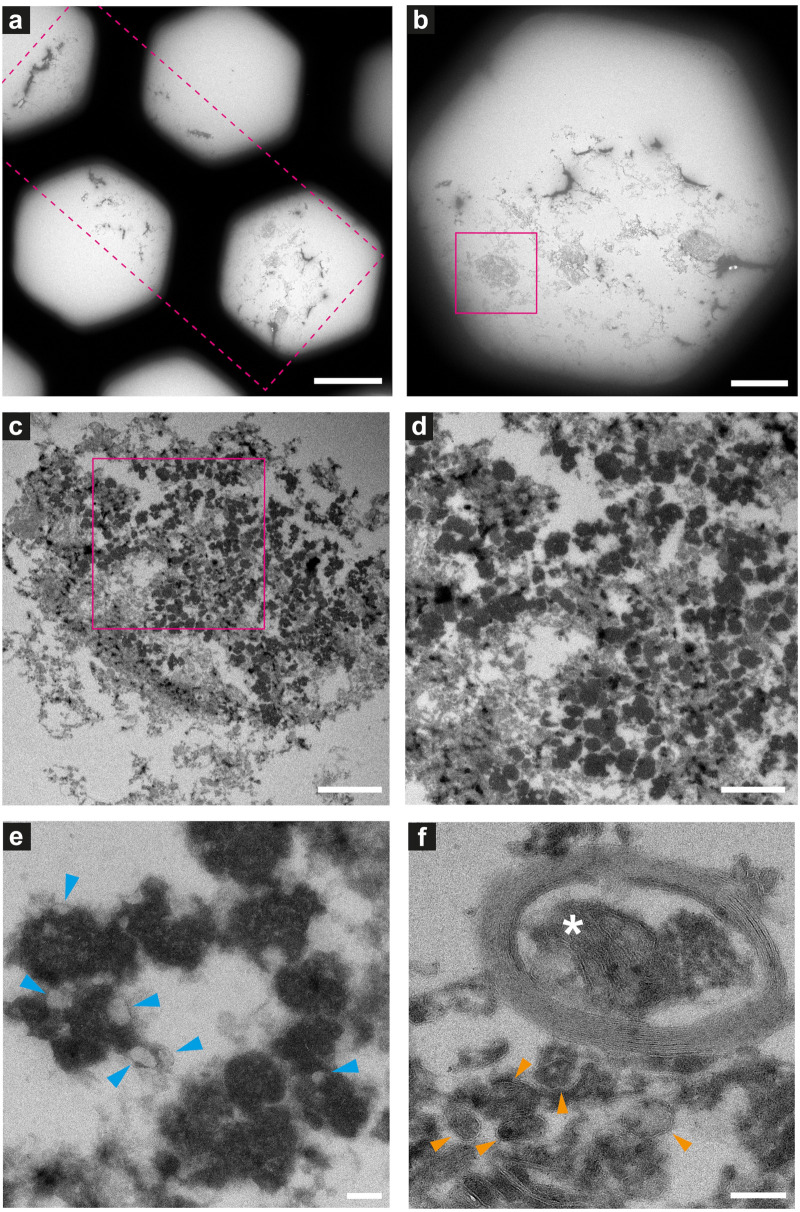
Electron microscopy of ultrathin tissue sections post cryo-PXCT imaging of non-demented control human brain. Sections were generated by cryo-ultramicrotomy of the tissue block of non-demented control human brain (Tomo 5, Table 1) and contrast-enhanced using uranyl acetate, following cryo-PXCT imaging. Images show progressively zoomed-in views of a neuromelanin containing cell and other higher-resolution cellular features. **(a)** Overview of the ultrathin tissue sections (pink dotted box) on the formvar-carbon support film of a hexagonal EM grid. **(b)** One neuromelanin-containing cell (pink box) visible in a section zoomed-in from panel **(a)**. **(c)** Zoomed-in view of the same neuromelanin-containing cell as shown in panel **(b)**, **(d)** Zoomed-in view of the neuromelanin as shown in the pink box in panel **(c)**, **(e)** Neuromelanin granules (dark dense blobs) and typically associated lipid globules (blue arrowheads) are visible at higher magnification and resolution, **(f)** A mitochondrion (white asterix) is visible within the cross-section of a myelinated axon in which the individual membranes of the myelin are clearly visible, as well as individual membranes of other vesicles and features within the tissue (orange arrowheads). Scale bars: **(a)** = 80 μm; **(b)** = 30 μm; **(c)** = 3 μm; **(d)** = 2 μm; **(e,f)** = 200 nm.

**FIGURE 9:**
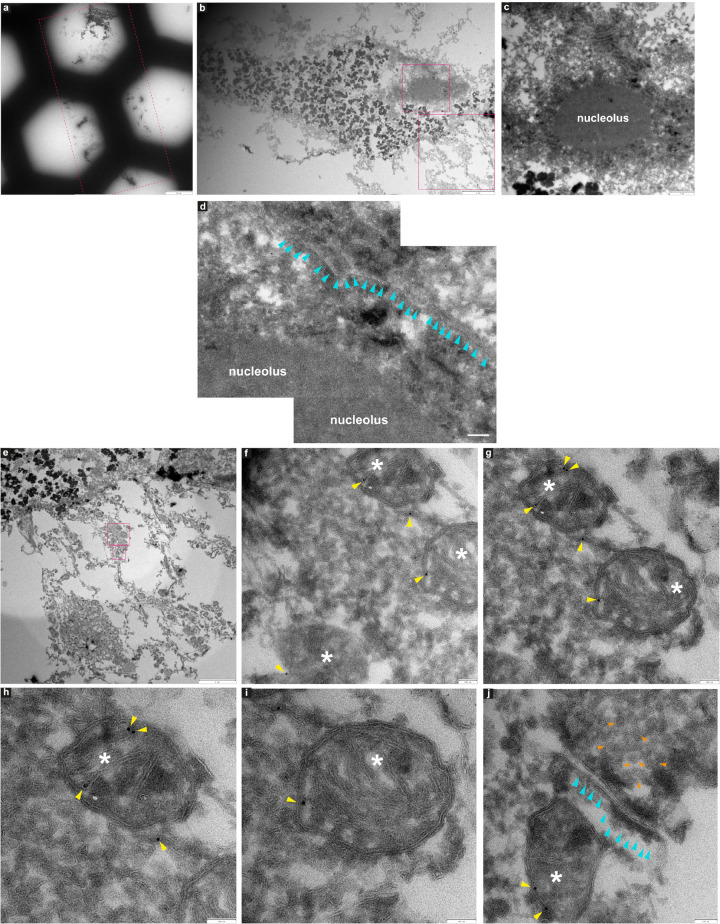
Immuno-electron microscopy of ultrathin tissue sections post cryo-PXCT imaging of non-demented control human brain. After cryo-PXCT imaging, sections were generated by cryo-ultramicrotomy of the tissue block of non-demented control human brain (Tomo 5, Table 1) followed by immunogold labeling using anti-VDAC1 (mitochondrial membrane marker), standard uranyl acetate contrast enhancement, and electron microscopy. Images **(a–c)** show progressively zoomed-in views of a neuromelanin containing cell. **(a)** Overview of the ultrathin tissue sections (pink dotted box) on the formvar-carbon support film of a hexagonal EM grid. **(b)** One neuromelanin-containing cell visible in a section zoomed-in from panel **(a)**. Neuromelanin granules are the dark dense globules. **(c)** Zoomed-in view of the nucleus [top pink box in panel **(b)**] including the clearly visible nucleolus of the same neuromelanin-containing cell, **(d)** Zoomed-in view of a part of the same nucleolus within the nucleus of the neuromelanin-containing cell, in which the nuclear membrane (aqua arrowheads) is preserved and clearly visible. **(e)** Zoomed-in view of the region as shown in the bottom pink box depicted in panel **(b)**, directly underneath the neuromelanin-containing cell, **(f)** three mitochondria (white asterix) zoomed-in from the top pink box as shown in panel **(e)**, with anti-VDAC1 (outer mitochondrial membrane marker) immunogolds (yellow arrowheads) visible as expected on their periphery. **(g)** View of two of the same mitochondria as shown in panel **(f)** in which more immunogolds (yellow arrowheads) can be visible at the top of the top-most mitochondrion. **(h)** Zoomed-in view of the top-most mitochondrion (white asterix) as shown in panel **(g)** where four immunogolds (yellow arrowheads) are visible. **(i)** Zoomed-in view of the bottom mitochondrion (white asterix) shown in panel **(g)** where one immunogold is visible. **(j)** Zoomed-in view of the synaptic cleft as shown in the bottom-most pink box shown in panel **(e)**, in which the membranes of the synaptic cleft (aqua arrowheads) are visible, a mitochondrion (white asterix) with two immunogolds (yellow arrowheads) visible on the post-synaptic side, and clusters of synaptic vesicles (orange arrowheads) visible on the presynaptic side. Scale bars: **(a)** = 60 μm; **(b)** = 4 μm; **(c)** = 1 μm; **(d)** = 4 μm; **(e)** = 3 μm; **(f–j)** = 100 nm.

**FIGURE 10:**
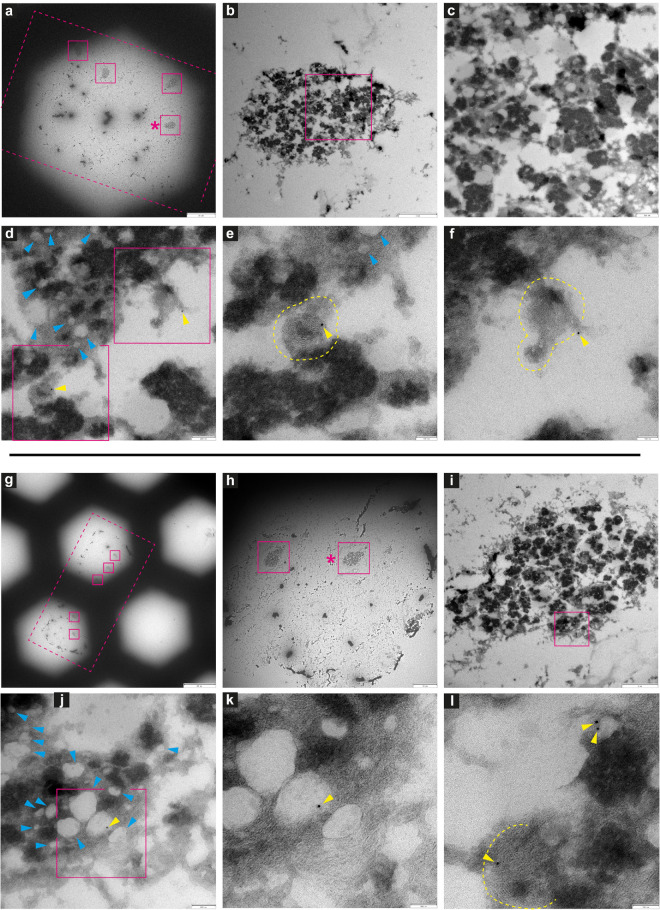
Immuno-electron microscopy of ultrathin tissue sections post cryo-PXCT imaging of Parkinson’s diseased human brain. After cryo-PXCT imaging, sections were generated by cryo-ultramicrotomy of the tissue block of Parkinson’s diseased human brain (Tomo 2, Table 1) followed by immunogold labeling, standard uranyl acetate contrast enhancement, and electron microscopy. Images in panels **(a–f)** and **(g–l)** correspond to different tissue sections: **(a–f)** immunogold-labeled using LB509 (anti-alpha synuclein), and **(g–l)** immunogold-labeled for p-aSyn (anti- phosphorylated alpha synuclein at S129). Images show progressively zoomed-in views of a neuromelanin containing cell from each tissue section. **(a)** Overview of the ultrathin tissue sections (pink dotted box) on the formvar-carbon support film of a hexagonal EM grid, with neuromelanin-containing cells visible (pink solid boxes). **(b)** Zoomed-in view of neuromelanin-containing cell shown in pink box (asterix) in panel **(a)**, and progressively zoomed-in view in panel **(c)** where lipid globules (pale gray globules) are clustered with neuromelanin granules (darker globules) as typically expected. **(d)** Zoomed-in view within the neuromelanin-containing cell where lipid globules are more clearly visible (blue arrowheads) and immunogolds (yellow arrowheads) for LB509 (anti alpha-synuclein) are visible. **(e)** Zoomed-in view of the bottom-most pink box shown in panel **(d)**, and the **(f)** top-most pink box shown in panel **(d)**, in which immunogolds for LB509 (anti alpha-synuclein) are visible, both on lamellar multi-membranous structures. **(g)** Overview of the ultrathin tissue sections (pink dotted box) on the formvar-carbon support film of a hexagonal EM grid, with neuromelanin-containing cells visible (pink solid boxes). **(h)** Zoomed-in view of neuromelanin-containing cell shown in pink box (asterix) in panel **(g)**, and progressively zoomed-in view in panel **(i)** where lipid globules (pale gray globules) are clustered with neuromelanin granules (darker globules) as typically expected. **(j)** Zoomed-in view within the neuromelanin-containing cell where membrane-enclosed structures are visible (blue arrowheads) and immunogold (yellow arrowhead) is visible, **(k)** zoomed-in view of the region in pink box shown in panel **(j)**, where immunogold is visible at the edge of a vesicle. **(l)** Another zoomed-in view of the neuromelanin-containing cell in which an immunogold is visible on another lamellar multi-membranous structure (delimited by yellow dotted lines) and two immunogolds are visible at the edge of a vesicle. Scale bars: **(a)** = 30 μm; **(b)** = 3 μm; **(c)** = 500 nm; **(d)** = 200 nm; **(e,f)** = 100 nm; **(g)** = 80 μm; **(h)** = 10 μm; **(i)** = 2 μm; **(j)** = 200 nm; **(k,l)** = 100 nm.

Each tissue block was schematically divided into three zones, and ultra-microtomed sections (70 nm thin) were cut and collected onto TEM grids sequentially from top of the block to bottom of the block, and immunolabeled accordingly. EM grids at low magnification showed the general shape and position of the resulting ultrathin sections from control human brain (Figures 8a, 9a) and PD human brain (Figures 10a,g). The integrity of the tissue was as expected for cryo immunogold labeling (Tokuyasu, 1986; Peters et al., 2006), with the gaps and holes typical of the methodology when applied to non-densely packed tissues with high water content like brain.

Neuromelanin-containing cells, of high clinical relevance in PD, were the most prominent and easily identifiable features within the tissue sections (Figures 8–10), after using the cryo-PXCT tomographic maps to navigate to the appropriate region in which these cells were originally found. By scrolling up and down through the corresponding cryo-PXCT reconstructed tomogram, we could determine the appropriate region (top, middle, bottom; edge, center) of the imaged cube in which the cells where located, which allowed to define the immunogold-stained tissue section(s) that would be most relevant to subsequently image. One neuromelanin-containing cell (Figures 8b,c) from a non-immunogold stained tissue section (Figure 8a) from a non-demented control human brain donor is shown to display clear neuromelanin-containing organelles in which the lipid vesicle component is clearly preserved amongst the typically dark, dense globules representing neuromelanin-containing organelles (Zucca et al., 2018) (Figures 8d,e, blue arrowheads). The dark appearance of the globules by electron microscopy (Figures 8c–e) is attributable to the naturally high metal content of such structures, while the dark appearance typically observed by brightfield light microscopy is attributable to the fact that these are pigmented akin to melanin in skin tissue. Furthermore, mitochondria with clear cristae were also visible within the tissue sections, one of which is shown enclosed within a clear axonal cross section (Figure 8F, white asterisk). The individual lipid layers of myelin sheath that compose the axon are also clearly visible, as well as the membranous profiles of vesicles (Figure 8f, orange arrowheads).

In another neuromelanin-containing cell (Figure 9b) shown in a control human brain tissue section (Figure 9a) that was immunogold-stained for a prominent mitochondrial porin antibody (VDAC1, Abcam ab14734), the nucleus and nucleolus are clearly visible (Figures 9c,d); furthermore, the nuclear membrane is also visible at higher magnification (Figure 9d, aqua arrowheads). Below the neuromelanin-containing cell (Figure 9e), several mitochondria were visualized, some of which are shown in Figures 9f–j (white asterix). Immunogolds (Figures 9f–j, yellow arrowheads) are visible at the edges of the mitochondria, where the VDAC1 porin protein is expected to be localized, being an outer mitochondrial membrane protein. Specificity is demonstrated by the lack of labeling in the tissue surrounding the mitochondria. This suggests that some protein antigenicity is retained even after cryo-PXCT imaging. Interestingly, a pool of synaptic vesicles (Figure 9j, orange arrowheads), synaptic cleft (aqua arrowheads), and a mitochondrion (white asterix) visible in the postsynaptic zone, were also clearly visible.

Several neuromelanin-containing cells (Figures 10a,b,g,h) could be localized in the appropriate EM tissue sections from the PD brain tissue samples, using the corresponding cryo-PXCT tomograms as a guide. In one tissue section (Figure 10a) immunogold-stained for LB509 (Abcam ab27766), an antibody that is routinely used to identify Lewy pathology as aSyn-immunopositive structures in PD, we observed that immunogolds localized to lamellar membranous structures (Figures 10e,f, yellow dotted lines) within the neuromelanin-containing organelles. These immunogolds were not visible in the background, suggesting a specificity to these structures. Similar to the control human brain tissue section, the quality of the tissue post cryo-PXCT imaging was sufficient to clearly resolve both the neuromelanin-containing organelles (Figures 10b–f,i–l, black, dark-contrast features) and their typical corresponding lipidic vesicle component known as lipid bodies (Zucca et al., 2018) (Figures 10d,e,j, blue arrowheads). In another tissue section (Figure 10g) immunogold-stained for p-aSyn (phospho S129, Abcam ab59264), we found immunogolds (yellow arrowheads) localized to the edge of the lipid bodies. Our results are in line with similar immunogold electron microscopy-based studies that show aSyn is localized in the neuromelanin (Zucca et al., 2018), similar to our result. Those researchers suggest that the process of neuromelanin synthesis that starts in the cytosol, may involve accumulation of aggregated and β-structured proteins, including aSyn (Zucca et al., 2018).

## Discussion

Our results using cryo-PXCT on unstained human brain tissue samples have revealed that several key cellular components can be resolved at the nanoscale, including myelinated axons, neuromelanin-containing cells and their nuclei, the nuclei of other brain cells such as glia, red blood cells and blood vessels within the brain. We have demonstrated that resolution is sufficient to visualize fine details including changes between the myelin sheaths of axons, particularly clarifying the ultrastructural nature of swellings within axons in this case. Not only can such structures be clearly visualized, but also with sufficient resolution (145–390 nm) providing a level of detail that allows us to clarify whether these swellings are occurring within the actual axon or within the myelin sheath composing the axon, suggestive of different biological mechanisms. We have found that such swellings, or dystrophic myelinated axons (DMAs), appear only within the PD human brain tissue samples as compared to the control brain samples that we have imaged; in this case, in the SNpc brain region that is highly clinically relevant to this disease.

As the phosphorylation of serine 129 of alpha synuclein (p-aSyn) is considered the dominant pathological modification in both familial and sporadic Lewy body diseases including PD, we have investigated the pathological relevance of the DMAs in this PD case using a phosphorylated alpha-synuclein antibody (p-aSyn, Ser129-P aSyn, 11A5, gift from Prothena) for fluorescent confocal microscopy, together with a Bielschowsky silver staining protocol (Uchihara, 2007) followed by multispectral imaging, on adjacent sections from same tissues as used for the X-ray/EM studies. By co-visualizing the nerve fibers with the Bielschowsky stain as well as p-aSyn-immunopositive Lewy neurites in adjacent tissue blocks to those collected for cryo-PXCT, we found that immunoreactivity overlapped partially or completely with swollen axons, demonstrating the presence of such pathologically relevant p-aSyn inclusions in these DMAs of the PD brain samples (Figure 7).

We have also shown that the majority of these DMAs occur within the myelin sheaths (Supplementary Figure 3 and Figure 6) corresponding to the cytoplasm of the parent oligodendrocyte, in contrast to the expectation that they would occur within the main passage of the axon corresponding to the neuronal cell itself. This suggests that processes in which the oligodendrocytes, or cells that produce the myelin sheath, are involved in the pathology of PD. The number of reports on the role of oligodendroglial cells in neurodegeneration has increased substantially over recent years (Ettle et al., 2016). In addition to their well-known role of producing myelin that mediates action potential conduction and communication between neurons, oligodendrocytes also provide trophic support for axonal and neuronal maintenance. Typically, oligodendrocytes are implicated in diseases such as multiple systems atrophy (MSA), amyotrophic lateral sclerosis (ALS), and more recently, Alzheimer’s disease (AD) (Chevalier-Larsen and Holzbaur, 2006). The aggregated cellular material that we observe in the oligodendrocyte cytoplasm composing the myelin sheaths (Figures 3b,d, 4, 6 and Supplementary Figures 1, 2), may indeed correspond to a pathological accumulation, one likely involving aSyn according to our in-parallel confocal microscopy datasets that reveal aSyn-immunopositive aggregates co-localizing with swollen axons (Figure 7).

This pathological accumulation within these swellings appeared very granular (Figure 4 and Supplementary Figures 1, 2), which we attribute to the presence of clustered vesicles. Indeed, correlative light and electron microscopy and tomography, and parallel studies using multi-labeling super resolution light microscopy, have recently shown a high lipid content and a crowding of vesicular structures including lysosomes and autophagosomes within both Lewy bodies and dystrophic axons corresponding to be Lewy neurites based on immunohistochemical staining (Shahmoradian et al., 2017a). Multiple studies have shown a potential link between disturbances in myelin integrity, myelin breakdown and axonal damage as an early event in the onset of neurodegenerative diseases including PD, AD, and Huntington’s disease (HD), using magnetic resonance imaging (MRI) (Bartzokis, 2004; Rosas et al., 2006). Furthermore, the occurrence of heightened tissue iron levels complexed with ferritin (produced in the brain mainly by oligodendrocytes) in PD, AD, and HD (Bartzokis et al., 2004, 2007; Todorich et al., 2009) is known to increase the concentration of reactive oxygen species (Puntarulo, 2005) that can initiate changes in the proteins’ tertiary structure, leading to aggregation that is associated with neurodegeneration. High tissue iron is considered as a risk factor in developing neurodegenerative disease, with oligodendrocytes playing a key role (Grune et al., 2004; Doorn et al., 2014; Ward et al., 2014).

Considering that myelin represents a vital factor for human brain connectivity, is profoundly evolved in humans compared to non-human primates and other mammals, and progressively declines in the aging human brain, oligodendrocyte dysfunction would logically contribute to the vulnerability of the human brain in regard to neurodegenerative diseases. We have demonstrated that cryo-PXCT is a useful tool to simultaneously visualize several axons in a continuous volume, currently limited to ∼(100 μm)^3^, without the addition of any stain, allowing the detection of subtle changes in axonal ultrastructure and enabling us to distinguish between types of swellings and furthermore the extent to which the axon is myelinated: thin myelin sheaths which are more frequently associated with neurodegeneration, as compared to thicker myelin sheaths which are more frequently associated with a healthier state (Braak et al., 2006b; Braak and Del Tredici, 2016). While cryo-PXCT would hence be useful to investigate diseases in which neuronal, oligodendrocytes and axonal degeneration are majorly implicated, our unexpected finding of axonal swellings directly arising within the myelin sheaths originating from the parent oligodendrocyte rather than within the axoplasm of the associated neuron from the human postmortem PD brain, brings forth the question of whether other neurodegenerative diseases displaying axonal swellings may also involve oligodendrocytes in a similar manner. Such an observation would have implications for disease pathogenesis and warrant closer investigation using complementary techniques.

Recognizing and differentiating abnormal features within the axon as opposed to those within the myelin sheath that encase the axon, is a crucial step toward pinpointing the underlying physiological and pathological processes. For example, abnormalities within the axon rather than the myelin sheath would implicate a cellular process that is specific to the parent neuron from which the axon extends. Abnormalities within the myelin sheath itself would correspond to pathologically relevant processes that are occurring within the parent oligodendrocyte cell from which the myelin sheath is produced. More specifically, each individual wrapping of myelin sheath around an axon protruding from a neuron, also contains its own cytoplasm that belongs to the oligodendrocyte cell producing that myelin sheath (Simons and Nave, 2015). Since oligodendrocyte cells are different in both composition and function as compared to neurons, abnormalities arising in their cytoplasm are important to distinguish from abnormalities in the neuronal cytoplasm, and would refer to a pathological process attributable to a different – either separate or concerted – cellular mechanism (Bradl and Lassmann, 2010).

Furthermore, we have demonstrated for the first time that after cryo-PXCT imaging, protein antigenicity is preserved, as demonstrated here by subsequent ultramicrotomy, immunogold labeling, and correlative electron microscopy (Figures 8–10), revealing the ultrastructure of cellular features including mitochondria, myelinated axons, synaptic clefts and the typical associated protein densities, synaptic vesicles, neuromelanin-containing organelles, nuclear membrane, nucleus and nucleolus, and various lipid vesicles. This demonstration has important implications for investigations which would greatly benefit from first generating a large map of complex, hierarchical multi-component features – typical to the crowded environment of biological tissues – by using cryo-PXCT, and subsequently imaging at higher resolution using correlative electron microscopy of target structures localized in the cryo-PXCT tomograms.

In general, X-ray ptychography has been shown to provide resolutions as high as 20 nm in 2D projections of single biological cryo-preserved cells (Deng et al., 2017). There is potential for increasing the resolution in large tissue volumes such as the ones investigated here, as there are studies supporting that biological tissue could withstand doses up to 1e9 Grays, which is about 2 orders of magnitude more than the dose deposited by PXCT in our work, while preserving features at length scales well below 100 nm (Howells et al., 2009). The development of diffraction-limited storage rings (Eriksson et al., 2014) and other improvements in instrumentation will allow to increase the coherent flux required for these experiments and thereby the spatial resolution could be increased up to the limit imposed by radiation damage within practical measurement times.

We have shown that cryo-PXCT is a useful tool for visualizing several features in both control and diseased postmortem human brain tissue samples, and for facilitating detection of subtle ultrastructural differences amongst structures that continuously span the examined tissue volumes, especially axons in this case. Evidence suggests that changes to axonal ultrastructure are considered as one of the early events in neurodegeneration, thereby justifying closer nanoscale-based investigations. For example, the appearance of neurite swellings marks an early event in neuritic degeneration in Parkinson’s diseased patient-derived neurons (Kouroupi et al., 2017), and dystrophic axons and alterations in axonal transport induced by overexpression of mutant alpha synuclein (p.A53T) in rats are known to precede neuronal loss (Chung et al., 2009).

Although our cryo-PXCT study generated five datasets from a single control aged human brain and four datasets restricted to a single age-matched PD brain donor, the unexpected finding of expansions and aggregations in myelin sheaths corresponding to the cytoplasm of the parent oligodendrocytes in the PD brain suggests a closer investigation of such processes in PD and other neurodegenerative conditions. Our results also prove cryo-PXCT as an appropriate tool for imaging such phenomena and related ultrastructural changes at the nanoscale. More remains to be clarified on samples taken from more patients, how such dense cytoplasmic aggregations arise within the parent oligodendrocytes, their specific relation with the dominant pathological form of alpha synuclein (phosphorylated Ser-129) (Anderson et al., 2006), and why they occur at specific points along the axon. Investigating across PD brains of different disease stages (Braak stages) and quantifying both the incidence and heterogeneity at the nanoscale of such DMAs may also prove to be useful in uncovering new aspects of the disease progression. Furthermore, our results warrant further investigations in other neurodegenerative diseases in which oligodendrocytes and axonal abnormalities are primarily involved, to yield a better understanding of subtle nanoscale changes that occur in different disease states and stages.

Here we have shown for the first time that nanoscale label-free imaging of diseased human brain tissues using hard X-rays can visualize several ultrastructural features and provide insight to pathologically relevant processes spanning continuous volumes. Our successful demonstration of downstream electron microscopy, and immunogold labeling for electron microscopy, post cryo-PXCT imaging on human brain tissue is anticipated to finally open the doors for clarifying the identity and ultrastructure of nanoscale biological features in large volume X-ray tomographic data. While the imaging rate of cryo-PXCT is currently comparable to destructive methods, with synchrotron upgrades occurring worldwide according to the multi-bend achromat and additional beamline improvements we expect an increase in coherent photon flux by up to four orders of magnitude, and thus a dramatic increase in imaging rate and/or resolution (Holler et al., 2017a). This will allow increasing the number of samples studied for a wider investigation.

## Methods

### Human Postmortem Brain Tissue Samples

Post-mortem brain tissue samples from *Donors A* and *B* (Table 2) with clinical diagnosis PD with dementia (PDD) and brain tissue samples from non-demented patients as controls (*Donors C-E*, Table 2) were obtained from the Netherlands Brain Bank (NBB^1^; Table 2) and the Normal Aging Brain Collection (Dept. Anatomy and Neurosciences, VUmc), respectively. Tissues were collected using a rapid autopsy protocol (NBB). Brain tissues from *Donors B* and *D* (Table 2) were used for cryo-PXCT and electron microscopy studies, while tissues from all donors were used for optical microscopy studies.

All protocols of the Netherlands Brain Bank (NBB), Netherlands Institute for Neuroscience, Amsterdam (open access; see text footnote 1), and of the Normal Aging Brain Collection (NABC), VU University Medical Center, Amsterdam, were approved by the Medical Ethical Committee (METC), VU University Medical Center, Amsterdam, the Netherlands. For brain samples and/or bio samples obtained from the NBB, all material has been collected from donors for or from whom a written informed consent for a brain autopsy and the use of the material and clinical information for research purposes was obtained by the NBB. For brain samples obtained from NABC, all material has been collected from donors for or from whom a written informed consent for a autopsy and the use of the material and clinical information for teaching and research purposes was obtained by the department of Anatomy and Neurosciences, VUmc, the Netherlands. For samples from both brain banks, detailed neuropathological and clinical information was made available, in compliance with local ethical and legal guidelines, and all protocols were approved by the local institutional review board.

At autopsy, 0.5 cm-thick adjacent brain slices of the SNpc were collected. Cubes of ∼1–2 mm^3^ of the ventral part of the SNpc were dissected and fixed for 6 h in a mixture of 2% paraformaldehyde/2.5% glutaraldehyde in 0.15 M cacodylate buffer with 2 mM calcium chloride, pH 7.4 and then washed with PBS. The PD brain donor fulfilled the United Kingdom Parkinson’s Disease Society Brain Bank (UK-PDSBB) clinical diagnostic criteria for PD (Emre et al., 2007). Neuropathological evaluation was performed on 7 μm formalin-fixed paraffin-embedded sections collected from multiple brain regions according to the guidelines of BrainNet Europe.

As is routine for such brain donors, staging of Alzheimer’s disease was evaluated according to the Braak criteria for NFTs (Braak et al., 2006a), CERAD criteria adjusted for age and Thal criteria (Thal et al., 2006). The presence and topographical distribution of aSyn (monoclonal mouse anti-human-α-synuclein, clone KM51, Monosan; Supplementary Figure 1) was rated according to Braak’s staging scheme for aSyn (Braak et al., 2004) and a modified version of McKeith’s staging system for aSyn (i.e., brainstem, limbic system, amygdala-predominant or neocortical (McKeith et al., 2005).

### Safety Considerations for Tissue Handling

All tools/surfaces coming in contact with the chemically fixed, postmortem human brain tissues were sterilized with a mixture of 2% sodium dodecyl sulfate (SDS) and 1% acetic acid, for sterilization against potential pathogenic agents (Rutala and Weber, 2010). For delicate parts such as the fine diamond tips of the Diatome^TM^ knives, 50% ethanol – as recommended – was used.

### Tissue Preparation for Cryo-PXCT Imaging

Tissues were prepared as previously described for mouse brain (Shahmoradian et al., 2017b) with some alterations regarding the final trimmed sample shape and more details provided herein, also shown in a workflow (Supplementary Figure 6). The fixed tissue was sectioned using a Vibratome into 60 μm-thick slices, kept at 4°C in glass scintillator vials with tight rubber seals in 0.15M cacodylate buffer. PBS (without calcium or magnesium) can be substituted. Circular regions were biopsy-punched out from the tissue in the neuromelanin-rich areas using a Harris Uni-Core biopsy punch tool (diameter 1.20 mm) on a Harris Cutting Mat. Punched-out pieces were placed in cryoprotectant (1.2M sucrose with 15% polyvinylpyrrolidone) in small plastic vials, rotating at 4°C for 2–3 weeks. Prior to cryo-ultramicrotomy, tissue pieces were checked to ensure that all sunk to the bottom of the tubes, indicating full penetration of cryoprotectant to the tissue. Tissue pieces were kept in the tubes on ice while preparing the OMNY pins (Holler et al., 2017b) in a method as previously described (Shahmoradian et al., 2017b).

On the day of cryo-trimming, cryo-knives (Diatome^TM^ 45° diamond trim knife and 90° diamond trim knife) were loaded and the cryo chamber was cooled to −90°C. OMNY pins were slightly shaved down to an appropriate height as previously described. For dissection, pieces of biopsy-punched tissue were placed in droplets of cryo-protectant on a black plastic block on ice; a black piece of paper glued to the other side of a petri dish, on ice, could also be used. This black plastic block (Leica) is typically used for mounting of pins for cryo ultramicrotomy Tokuyasu technique. A Microfeather 30° ophthalmological scalpel was used to cut the pieces (in cryoprotectant) into four quadrants, further cut into 8 “pie pieces” in total. The sample was held in place using fine electronic-grade tweezers during this cutting.

The leg of one of the tweezers was used to position one “pie-piece” of tissue onto the tip of a shaved OMNY pin (Shahmoradian et al., 2017b) positioned in a small aluminum cube adaptor. Ethanol (70%) was used to continuously clean the tweezers to prevent hardening/sticking due to the cryoprotectant. A clean 200 μl pipette tip was used to gently position the piece of tissue straight and centered onto the OMNY pin. The pipette tip did not stick as much to the tissue as the metal tweezers. The OMNY pin and adaptor was then placed into the cryo chamber at −90°C and kept therein for 1 h. This is a “slow-freezing” technique typical for cryo-immunogold electron microscopy (Tokuyasu, 1986; Peters et al., 2006). This procedure was repeated for mounting all “pie pieces” of tissue from two biopsy-punched pieces of tissue, onto multiple OMNY pins. The small aluminum cube adaptors, holding the OMNY pin on which each tissue piece was previously “glued” using the extra cryoprotectant (liquid at room temperature, then hardened while transferred and left in the cryo ultramicrotome chamber at −90°C), were then firmly clamped into the standard clamping chuck of the Leica cryo ultramicrotome for subsequent trimming into a skyscraper-type shape of approximately 100 μm in X-Y-Z dimensions using a Trim90 (Diatome^TM^) diamond knife with a 90° cutting angle. Samples were stored in liquid nitrogen until imaging by cryo-PXCT. Safety considerations were performed according to section “Safety Considerations for Tissue Handling.”

### Cryo-PXCT Data Collection and Tomogram Reconstruction

Cryo-PXCT measurements were carried out at the cSAXS beamline at the Swiss Light Source, Paul Scherrer Institut (PSI), Switzerland. Nine tomograms (four of Parkinson’s diseased human brain and five of non-demented control human brain) were obtained under cryogenic conditions (−180°C) using OMNY (Holler et al., 2018). A general description of the experimental setup is provided as follows. Samples were mounted on customized sample pins (Holler et al., 2017b) and imaged at a photon energy of 6.2 keV, defined by a double crystal Si (111) monochromator. The illumination on the sample was defined by the combination of a 50 μm-diameter central stop, a coherently illuminated 220 μm-diameter Fresnel zone plate (FZP) with an outer-most zone width of 60 nm, and a 30 μm-diameter order sorting aperture. The FZP was fabricated by the Laboratory for Micro and Nanotechnology, Paul Scherrer Institut. The focal distance was 66 mm while the sample was placed 2.4 mm downstream the focus to give an illumination of around 8 um in diameter on the sample. For each ptychographic projection, the scanning followed a Fermat spiral pattern (Huang et al., 2014) with an average step size of 2.6 micron. For each scanning position, a diffraction pattern was collected 7.3 m downstream of the sample with an EIGER (Guizar-Sicairos et al., 2014) detector and exposure time of 0.1 s. Projections were taken from 0 to 180 degrees. The field of view and the number of projections for each tomogram are detailed in Table 1.

Ptychographic reconstructions were obtained through the difference map algorithm (Thibault et al., 2008) followed by maximum likelihood algorithm (Thibault and Guizar-Sicairos, 2012) using software PtychoShelves (Wakonig et al., 2020). For each diffraction pattern an area of 500 by 500 pixels was used for the reconstruction, giving an image pixel size of approximately 40 nm. 2D projections were aligned (Guizar-Sicairos et al., 2011, 2015) to generate 3D tomograms based on modified filtered back projection (Guizar-Sicairos et al., 2011). The grayscale in the tomograms correspond to absolute electron density (Diaz et al., 2012). Image resolution was estimated by FSC (Van Heel and Schatz, 2005). The number of photons incident on the sample for one projection fell in the range of 2.5 × 10^6^ to 4.5 × 10^6^ photons/μm^2^. X-ray doses exposed to the frozen brain samples were estimated using absorption coefficients of water with attenuation length of 451 μm and density 1,000 kg/m^3^. Estimated resolutions and X-ray doses can be found for each measurement in Table 1. Safety considerations were performed according to section “Safety Considerations for Tissue Handling.”

### Immuno-Electron Microscopy After Cryo-PXCT Imaging

Tissue blocks corresponding to Tomo 5 (Control human brain) and Tomo 2 (Parkinson’s diseased human brain) were selected for downstream cryo-ultramicrotomy and immunogold labeling followed by imaging by electron microscopy (Supplementary Figure 5). Tissue blocks were mounted into the cryo ultramicrotome chamber and sectioned at −100°C, typical for cryo-immunogold labeling for electron microscopy (Tokuyasu, 1986; Peters et al., 2006). Sections of 70 nm thickness were created using a sectioning speed of 0.2 mm/s with a Diatome^TM^ “Cryo35” knife, without use of the static ionizer. Sections were picked up from surface of the knife using a Diatome^TM^ “Perfect Loop” with a droplet of solution prepared by adding 2.3 M sucrose in phosphate buffer to 2% methylcellulose in distilled water with a ratio 3:1, and transferred to the surface of a hexagonal 200-mesh gold EM grid using a technique as previously described (Peters et al., 2006).

Calcium-/magnesium-free PBS were used to wash sections from both control human brain and Parkinson’s diseased human brain tissue blocks to remove pick-up solution (three times for 2 min each). After washing, those sections were inactivated free aldehyde group by incubating with 50 mM glycine in calcium-/magnesium-free PBS for 15 min and were then blocked hydrophobic areas using AURION^TM^ Blocking Solution for Goat antibody Gold Conjugated (product code 905.002) for 30 min. After washing by calcium-/magnesium-free PBS buffer containing 0.1% AURION^TM^ BSA-c (product code 900.099), they were immunolabeled using the following primary antibodies: 1 μg/ml of anti-VDAC1 (mitochondrial porin antibody, Abcam ab14734), 2 μg/ml of anti-LAMP1 antibody (lysosomal marker, Abcam ab24170), 5 μg/ml of anti-alpha-synuclein (LB509, Abcam ab27766), or 10 μg/ml of anti- phosphorylated alpha-synuclein (S129, Abcam ab59264). Sections on each EM grid were subject to only 1 kind of antibody each (no multiple labeling). Antibodies were diluted in a calcium-/magnesium-free PBS buffer containing 0.1% AURION^TM^ BSA-c. Sections on the EM grids were incubated for 1 h at room temperature with the primary antibodies.

After primary antibody incubation, they were washed 6 times, 5 min each with calcium-/magnesium-free PBS solution containing 0.1% AURION^TM^ BSA-c. Secondary immunogolds (10 nm diameter, AURION^TM^ ImmunoGold reagents) were incubated for 90 min at room temperature, then washed with calcium-/magnesium-free PBS containing 0.1% AURION^TM^ BSA-c, followed by additional wash by calcium-/magnesium-free PBS. Afterward, sections on grids were postfixed by 2% glutaraldehyde in calcium-/magnesium-free PBS for 5 min. To remove glutaraldehyde, grids were washed extensively with calcium-/magnesium-free PBS, then deionized/distilled water. Subsequently, grids were additionally contrast-enhanced using 4% neutral uranyl acetate, which was prepared by mixing 4% uranyl acetate and 0.3M oxalic acid and was adjusted to pH 7 by 25% ammonium hydroxide. A solution of 0.4% uranyl acetate in 2% methylcellulose was then used for further contrast-enhancement (on ice). EM grids were then imaged at room temperature using an FEI T12 (Thermo Fisher Scientific, United States) operated at 120 kV. Electron micrographs were recorded on a 4096 × 4096 pixel F416 CMOS camera (TVIPS GmbH, Germany). Safety considerations were performed according to section “Safety Considerations for Tissue Handling.”

### 3D Color Segmentation and Statistical Analysis of Dystrophic Myelinated Axons (DMAs)

Three-dimensional visualization and color segmentation was performed using commercial software Avizo 9.2.0 (Thermo Scientific). The images were imported into Avizo software and the threshold of the colormap was adjusted appropriately. The features of interest including blood vessels, nuclei, red blood cells and myelinated axons were segmented semi-automatically with the use of the “Brush” and “Interpolate” tools. The neuromelanin-containing organelles that appear as dark, dense globules could be segmented by “Threshold” tool. The masking value was adjusted until all neuromelanin-containing organelles were masked and identified precisely, then were selected and assigned by “Select Masked Material” tool for all slices. Unexpected selected regions not corresponding to neuromelanin were deselected semi-automatically afterward by using “Brush” and “Interpolate” tools.

After all structures were segmented and registered to their appropriate materials (blood vessel, myelinated axon, etc.), they were smoothed independently by locking other materials. Afterward, smoothed materials were exported into individual data objects from the Labels dataset by the “Arithmetic” function and surfaces were then generated for visualization. Myelinated axons, neuromelanin, nuclei, red blood cells were visualized by the “Shaded” draw style while blood vessels and the swollen part of the myelinated axons were visualized by “Transparent” draw style to reveal their inside contents. This label separation process allowed us to visualize the surface of single material without disturbing others. Afterward, snapshots of 3D color-rendered surfaces and movies were created for presentation.

### Optical Microscopy Data Collection

Experiments were performed on 10 and 20 μm-thick formalin-fixed paraffin-embedded sections of the midbrain containing the SNpc, from 2 PD patients and 2 non-neurological control subjects, including adjacent tissue sections from the same PD brain donor as used for the cryo-PXCT and EM studies (Table 2). First, an immunofluorescent staining was performed using a primary antibody directed specifically against Ser-129 p-aSyn (11A5, Prothena, 0.3 μg/ml, incubation overnight at 4°C), a secondary antibody coupled to an Alexa 594 fluophore (Molecular Probes; art. no. A21203; 1/400 diluted; incubation 2 h at RT), and DAPI (1 μg/ml). Afterward, a Bielschowsky silver staining protocol was performed, according to the protocol previously described (Litchfield and Nagy, 2001).

Sections were analyzed by brightfield and fluorescent microscopy, which was performed using a Leica DM5000B automated microscope (Leica Microsystems), equipped with a Nuance camera (Nuance 3.02, Perkin Elmer Inc) for multispectral imaging. Images were captured at wavelengths ranging from 440 to 540 nm (for DAPI), and ranging from 580 to 720 nm (for p-aSyn) with HC PL APO 40 × 1.30 NA and HC PL APO 63 × 1.40 NA – 0.60 oil objectives. The spectrum of autofluorescent signal was determined and removed from the images. Brightfield images were subsequently made at the same locations using the Nuance camera. In addition, additional brightfield images were captured using a Leica DFC450 camera with PL FLUOTAR 2.5 X/0.07 and HC PL APO 10 × 0.40 NA objectives.

## Data Availability Statement

The raw data supporting the conclusions of this article will be made available by the authors, without undue reservation.

## Ethics Statement

The studies involving human participants were reviewed and approved by the medical ethical committee of VU University Medical Center Amsterdam. The patients/participants of next of kin provided written informed consent for the use of clinical data and brain tissue for research purposes.

## Author Contributions

HT performed all color segmentations of the tomograms, completed quantification and measurements for all dystrophic axons in all of the diseased datasets, made all movies, helped to make the schematic model, and performed ultramicrotomy and immunogold labeling. IR assisted with ultramicrotomy and immunogold labeling. ET, AD, MG-S, and MH collected the cryo-PXCT data. ET primarily completed all of the tomographic reconstructions and made the descriptive table. MH, JR, AD, and MG-S developed the OMNY instrument and associated software. WB dissected postmortem human brain tissue and provided all tissue samples from NBB and NABCA to SS. WB supervised the stainings and analysis with optical microscopy. HS provided resources and contributed to electron microscopy. SS interpreted biological results. AL collected electron microscopy data with SS and JB. TM performed the combined Bielchowsky and immunofluorescence experiment. Analysis of optical microscopy was done by TM, AJ, and JB. WB and TM wrote text of optical microscopy section. SS planned the experiments, trained and supervised HT, prepared and cryo-trimmed all tissues for subsequent cryo-PXCT imaging, made the figures, and wrote the manuscript, with input from all authors.

## Conflict of Interest

The authors declare that the research was conducted in the absence of any commercial or financial relationships that could be construed as a potential conflict of interest.

## Footnotes

1 www.brainbank.nl

## References

[B1] Adalbert R. Nogradi A. Babetto E. Janeckova L. Walker S. A. Kerschensteiner M. (2009). Severely dystrophic axons at amyloid plaques remain continuous and connected to viable cell bodies. *Brain* 132 402–416. 10.1093/brain/awn312 19059977

[B2] Anderson J. P. Walker D. E. Goldstein J. M. de Laat R. Banducci K. Caccavello R. J. (2006). Phosphorylation of Ser-129 is the dominant pathological modification of alpha-synuclein in familial and sporadic Lewy body disease. *J. Biol. Chem.* 281 29739–29752. 10.1074/jbc.M600933200 16847063

[B3] Bartels M. Krenkel M. Cloetens P. Möbius W. Salditt T. (2015). Myelinated mouse nerves studied by X-ray phase contrast zoom tomography. *J. Struct. Biol.* 192 561–568. 10.1016/j.jsb.2015.11.001 26546551

[B4] Bartzokis G. (2004). Age-related myelin breakdown: a developmental model of cognitive decline and Alzheimer’s disease. *Neurobiol. Aging* 25 5–18. 10.1016/j.neurobiolaging.2003.03.001 14675724

[B5] Bartzokis G. Lu P. H. Tishler T. A. Fong S. M. Oluwadara B. Finn J. P. (2007). Myelin breakdown and iron changes in Huntington’s disease: pathogenesis and treatment implications. *Neurochem. Res.* 32 1655–1664. 10.1007/s11064-007-9352-7 17484051

[B6] Bartzokis G. Tishler T. A. Shin I. S. Lu P. H. Cummings J. L. (2004). Brain ferritin iron as a risk factor for age at onset in neurodegenerative diseases. *Ann. N. Y. Acad. Sci.* 1012 224–236. 10.1196/annals.1306.019 15105269

[B7] Braak H. Alafuzoff I. Arzberger T. Kretzschmar H. Del Tredici K. (2006a). Staging of Alzheimer disease-associated neurofibrillary pathology using paraffin sections and immunocytochemistry. *Acta Neuropathol.* 112 389–404. 10.1007/s00401-006-0127-z 16906426 PMC3906709

[B8] Braak H. Del Tredici K. (2016). Potential pathways of abnormal tau and alpha-synuclein dissemination in sporadic alzheimer’s and parkinson’s diseases. *Cold Spring Harb. Perspect. Biol.* 8:a023630. 10.1101/cshperspect.a023630 27580631 PMC5088528

[B9] Braak H. Ghebremedhin E. Rub U. Bratzke H. Del Tredici K. (2004). Stages in the development of Parkinson’s disease-related pathology. *Cell Tissue Res.* 318 121–134. 10.1007/s00441-004-0956-9 15338272

[B10] Braak H. Rub U. Schultz C. Del Tredici K. (2006b). Vulnerability of cortical neurons to Alzheimer’s and Parkinson’s diseases. *J. Alzheimers Dis.* 9 35–44. 10.3233/JAD-2006-9S305 16914843

[B11] Bradl M. Lassmann H. (2010). Oligodendrocytes: biology and pathology. *Acta Neuropathol.* 119 37–53. 10.1007/s00401-009-0601-5 19847447 PMC2799635

[B12] Burke R. E. O’Malley K. (2013). Axon degeneration in Parkinson’s disease. *Exp. Neurol.* 246 72–83. 10.1016/j.expneurol.2012.01.011 22285449 PMC3340476

[B13] Chevalier-Larsen E. Holzbaur E. L. (2006). Axonal transport and neurodegenerative disease. *Biochim. Biophys. Acta (BBA) Mol. Basis Dis.* 1762 1094–1108. 10.1016/j.bbadis.2006.04.002 16730956

[B14] Chung C. Y. Koprich J. B. Siddiqi H. Isacson O. (2009). Dynamic changes in presynaptic and axonal transport proteins combined with striatal neuroinflammation precede dopaminergic neuronal loss in a rat model of AAV alpha-synucleinopathy. *J. Neurosci.* 29 3365–3373. 10.1523/JNEUROSCI.5427-08.2009 19295143 PMC2693917

[B15] de Castro Fonseca M. Araujo B. H. S. Dias C. S. B. Archilha N. L. Neto D. P. A. Cavalheiro E. (2018). High-resolution synchrotron-based X-ray microtomography as a tool to unveil the three-dimensional neuronal architecture of the brain. *Sci. Rep.* 8 1–13. 10.1038/s41598-018-30501-x 30104676 PMC6089932

[B16] Deng J. Vine D. J. Chen S. Jin Q. Nashed Y. S. Peterka T. (2017). X-ray ptychographic and fluorescence microscopy of frozen-hydrated cells using continuous scanning. *Sci. Rep.* 7:445. 10.1038/s41598-017-00569-y 28348401 PMC5428657

[B17] Diaz A. Malkova B. Holler M. Guizar-Sicairos M. Lima E. Panneels V. (2015). Three-dimensional mass density mapping of cellular ultrastructure by ptychographic X-ray nanotomography. *J. Struct. Biol.* 192 461–469. 10.1016/j.jsb.2015.10.008 26470812

[B18] Diaz A. Trtik P. Guizar-Sicairos M. Menzel A. Thibault P. Bunk O. (2012). Quantitative x-ray phase nanotomography. *Phys. Rev.B* 85:020104 10.1103/PhysRevB.85.020104

[B19] Doorn K. J. Goudriaan A. Blits-Huizinga C. Bol J. G. Rozemuller A. J. Hoogland P. V. (2014). Increased amoeboid microglial density in the olfactory bulb of Parkinson’s and Alzheimer’s patients. *Brain Pathol.* 24 152–165. 10.1111/bpa.12088 24033473 PMC8029318

[B20] Dyer E. L. Gray Roncal W. Prasad J. A. Fernandes H. L. Gürsoy D. Andrade V. De, et al. (2017). Quantifying mesoscale neuroanatomy using X-ray microtomography. *eNeuro* 4:ENEURO.0195-17.2017. 10.1523/ENEURO.0195-17.2017 29085899 PMC5659258

[B21] Emre M. Aarsland D. Brown R. Burn D. J. Duyckaerts C. Mizuno Y. (2007). Clinical diagnostic criteria for dementia associated with Parkinson’s disease. *Mov. Disord.* 22 1689–1707. 10.1002/mds.21507 17542011

[B22] Eriksson M. van der Veen J. F. Quitmann C. (2014). Diffraction-limited storage rings – a window to the science of tomorrow. *J. Synchrotron. Radiat.* 21 837–842. 10.1107/S1600577514019286 25177975

[B23] Ettle B. Schlachetzki J. C. M. Winkler J. (2016). Oligodendroglia and myelin in neurodegenerative diseases: more than just bystanders? *Mol. Neurobiol.* 53 3046–3062. 10.1007/s12035-015-9205-3 25966971 PMC4902834

[B24] Fearnley J. M. Lees A. J. (1991). Ageing and Parkinson’s disease: substantia nigra regional selectivity. *Brain* 114 2283–2301. 10.1093/brain/114.5.2283 1933245

[B25] Fields R. D. (2014). Myelin formation and remodeling. *Cell* 156 15–17. 10.1016/j.cell.2013.12.038 24439366 PMC5017146

[B26] Friese M. A. (2016). Widespread synaptic loss in multiple sclerosis. *Brain* 139 2–4. 10.1093/brain/awv349 26747852

[B27] Galvin J. E. Uryu K. Lee V. M. Trojanowski J. Q. (1999). Axon pathology in Parkinson’s disease and Lewy body dementia hippocampus contains alpha-, beta-, and gamma-synuclein. *Proc. Natl. Acad. Sci. U.S.A.* 96 13450–13455. 10.1073/pnas.96.23.13450 10557341 PMC23968

[B28] García-Cabezas M. Á John Y. J. Barbas H. Zikopoulos B. (2016). Distinction of neurons, glia and endothelial cells in the cerebral cortex: an algorithm based on cytological features. *Front. Neuroanat.* 10:107. 10.3389/fnana.2016.00107 27847469 PMC5088408

[B29] Grune T. Jung T. Merker K. Davies K. J. (2004). Decreased proteolysis caused by protein aggregates, inclusion bodies, plaques, lipofuscin, ceroid, and ‘aggresomes’ during oxidative stress, aging, and disease. *Int. J. Biochem. Cell Biol.* 36 2519–2530. 10.1016/j.biocel.2004.04.020 15325589

[B30] Guizar-Sicairos M. Diaz A. Holler M. Lucas M. S. Menzel A. Wepf R. A. (2011). Phase tomography from x-ray coherent diffractive imaging projections. *Optics express* 19 21345–21357. 10.1364/OE.19.021345 22108985

[B31] Guizar-Sicairos M. Boon J. J. Mader K. Diaz A. Menzel A. Bunk O. (2015). Quantitative interior x-ray nanotomography by a hybrid imaging technique. *Optica* 2 259–266. 10.1364/OPTICA.2.000259

[B32] Guizar-Sicairos M. Johnson I. Diaz A. Holler M. Karvinen P. Stadler H.-C. (2014). High-throughput ptychography using Eiger: scanning X-ray nano-imaging of extended regions. *Optics Express* 22 14859–14870. 10.1364/OE.22.014859 24977581

[B33] Holler M. Diaz A. Guizar-Sicairos M. Karvinen P. Farm E. Harkonen E. (2014). X-ray ptychographic computed tomography at 16 nm isotropic 3D resolution. *Sci. Rep.* 4:3857. 10.1038/srep03857 24457289 PMC3900995

[B34] Holler M. Guizar-Sicairos M. Tsai E. H. R. Dinapoli R. Muller E. Bunk O. (2017a). High-resolution non-destructive three-dimensional imaging of integrated circuits. *Nature* 543 402–406. 10.1038/nature21698 28300088

[B35] Holler M. Raabe J. Diaz A. Guizar-Sicairos M. Wepf R. Odstrcil M. (2018). OMNY—A tOMography Nano crYo stage. *Rev. Sci. Instrum.* 89:043706 10.1063/1.5020247 29716370

[B36] Holler M. Raabe J. Wepf R. Shahmoradian S. H. Diaz A. Sarafimov B. (2017b). OMNY PIN-A versatile sample holder for tomographic measurements at room and cryogenic temperatures. *Rev. Sci. Instrum.* 88:113701 10.1063/1.4996092 29195351

[B37] Howells M. R. Beetz T. Chapman H. N. Cui C. Holton J. M. Jacobsen C. J. (2009). An assessment of the resolution limitation due to radiation-damage in x-ray diffraction microscopy. *J. Electron Spectros Relat. Phenomena* 170 4–12. 10.1016/j.elspec.2008.10.008 20463854 PMC2867487

[B38] Huang X. Yan H. Harder R. Hwu Y. Robinson I. K. Chu Y. S. (2014). Optimization of overlap uniformness for ptychography. *Optics Express* 22 12634–12644. 10.1364/OE.22.012634 24921380

[B39] Inglese M. Petracca M. (2013). Imaging multiple sclerosis and other neurodegenerative diseases. *Prion* 7 47–54. 10.4161/pri.22650 23117868 PMC3609050

[B40] Khimchenko A. Bikis C. Pacureanu A. Hieber S. E. Thalmann P. Deyhle H. (2018). Hard X−ray nanoholotomography: large−scale, label−free, 3D neuroimaging beyond optical limit. *Adv. Sci.* 5:1700694. 10.1002/advs.201700694 29938163 PMC6010902

[B41] Koch J. C. Bitow F. Haack J. d’Hedouville Z. Zhang J. N. Tonges L. (2015). Alpha-Synuclein affects neurite morphology, autophagy, vesicle transport and axonal degeneration in CNS neurons. *Cell Death Dis.* 6:e1811. 10.1038/cddis.2015.169 26158517 PMC4650722

[B42] Kotzbauer P. T. Giasson B. I. Kravitz A. V. Golbe L. I. Mark M. H. Trojanowski J. Q. (2004). Fibrillization of alpha-synuclein and tau in familial Parkinson’s disease caused by the A53T alpha-synuclein mutation. *Exp. Neurol.* 187 279–288. 10.1016/j.expneurol.2004.01.007 15144854

[B43] Kouroupi G. Taoufik E. Vlachos I. S. Tsioras K. Antoniou N. Papastefanaki F. (2017). Defective synaptic connectivity and axonal neuropathology in a human iPSC-based model of familial Parkinson’s disease. *Proc. Natl. Acad. Sci. U.S.A.* 114 E3679–E3688. 10.1073/pnas.1617259114 28416701 PMC5422768

[B44] Kremers G. J. Gilbert S. G. Cranfill P. J. Davidson M. W. Piston D. W. (2011). Fluorescent proteins at a glance. *J. Cell Sci.* 124 157–160. 10.1242/jcs.072744 21187342 PMC3037093

[B45] Kuan A. T. Phelps J. S. Thomas L. A. Nguyen T. M. Han J. Chen C.-L. (2020). Dense neuronal reconstruction through X-ray holographic nano-tomography. *Nat. Neurosci.* 10.1038/s41593-020-0704-9 [Epub ahead of print]. 32929244 PMC8354006

[B46] Kuusisto E. Parkkinen L. Alafuzoff I. (2003). Morphogenesis of Lewy bodies: dissimilar incorporation of alpha-synuclein, ubiquitin, and p62. *J. Neuropathol. Exp. Neurol.* 62 1241–1253. 10.1093/jnen/62.12.1241 14692700

[B47] Lam S. S. Martell J. D. Kamer K. J. Deerinck T. J. Ellisman M. H. Mootha V. K. (2015). Directed evolution of APEX2 for electron microscopy and proximity labeling. *Nat. Methods* 12 51–54. 10.1038/nmeth.3179 25419960 PMC4296904

[B48] Le Gros M. A. Clowney E. J. Magklara A. Yen A. Markenscoff-Papadimitriou E. Colquitt B. (2016). Soft X-ray tomography reveals gradual chromatin compaction and reorganization during neurogenesis in vivo. *Cell Reports* 17 2125–2136. 10.1016/j.celrep.2016.10.060 27851973 PMC5135017

[B49] Lee J. Y. Taghian K. Petratos S. (2014). Axonal degeneration in multiple sclerosis: can we predict and prevent permanent disability? *Acta Neuropathol. Commun.* 2:97. 10.1186/s40478-014-0097-7 25159125 PMC4243718

[B50] Litchfield S. Nagy Z. (2001). New temperature modification makes the Bielschowsky silver stain reproducible. *Acta Neuropathol.* 101 17–21. 10.1007/s004010000248 11194935

[B51] Manning C. F. Bundros A. M. Trimmer J. S. (2012). Benefits and pitfalls of secondary antibodies: why choosing the right secondary is of primary importance. *PLoS One* 7:e38313. 10.1371/journal.pone.0038313 22675541 PMC3365890

[B52] McKeith I. G. Dickson D. W. Lowe J. Emre M. O’Brien J. T. Feldman H. (2005). Consortium on, diagnosis and management of dementia with Lewy bodies: third report of the DLB Consortium. *Neurology* 65 1863–1872. 10.1212/01.wnl.0000187889.17253.b1 16237129

[B53] Mizutani R. Takeuchi A. Uesugi K. Suzuki Y. Osamura R. Y. Takekoshi S. (2009). Microtomographic analysis of neuronal circuits of human brain. *Cereb. Cortex* 20 1739–1748. 10.1093/cercor/bhp237 19915092

[B54] Mokso R. Cloetens P. Maire E. Ludwig W. Buffière J. Y. (2007). Nanoscale zoom tomography with hard x rays using Kirkpatrick-Baez optics. *Appl. Phys. Lett.* 90:144104 10.1063/1.2719653

[B55] Morell P. Norton W. T. (1980). Myelin. *Sci. Am.* 242 88–119. 10.1038/scientificamerican0580-88 6154973

[B56] Nave K. A. Werner H. B. (2014). Myelination of the nervous system: mechanisms and functions. *Annu. Rev. Cell Dev. Biol.* 30 503–533. 10.1146/annurev-cellbio-100913-013101 25288117

[B57] Pérez-Berná A. J. Rodríguez M. J. Chichón F. J. Friesland M. F. Sorrentino A. Carrascosa J. L. (2016). Structural changes in cells imaged by soft X-ray cryo-tomography during hepatitis C virus infection. *ACS Nano* 10 6597–6611. 10.1021/acsnano.6b01374 27328170

[B58] Peters P. J. Bos E. Griekspoor A. (2006). Cryo−immunogold electron microscopy. *Curr. Protocols Cell Biol.* 30 4.7.1–4.7.19. 10.1002/0471143030.cb0407s30 18228493

[B59] Peters P. J. Pierson J. (2008). Immunogold labeling of thawed cryosections. *Methods Cell Biol.* 88 131–149. 10.1016/S0091-679X(08)00408-1 18617032

[B60] Pfister B. Sanchez-Ferrer A. Diaz A. Lu K. J. Otto C. Holler M. (2016). Recreating the synthesis of starch granules in yeast. *eLife* 5:29 10.7554/eLife.15552.024 PMC5119888 27871361

[B61] Puntarulo S. (2005). Iron, oxidative stress and human health. *Mol. Aspects Med.* 26 299–312. 10.1016/j.mam.2005.07.001 16102805

[B62] Rosas H. D. Tuch D. S. Hevelone N. D. Zaleta A. K. Vangel M. Hersch S. M. (2006). Diffusion tensor imaging in presymptomatic and early Huntington’s disease: selective white matter pathology and its relationship to clinical measures. *Mov. Disord.* 21 1317–1325. 10.1002/mds.20979 16755582

[B63] Rutala W. A. Weber D. J. (2010). A society for healthcare epidemiology of, guideline for disinfection and sterilization of prion-contaminated medical instruments. *Infect Control Hosp. Epidemiol.* 31 107–117. 10.1086/650197 20055640

[B64] Saha A. R. Hill J. Utton M. A. Asuni A. A. Ackerley S. Grierson A. J. (2004). Parkinson’s disease alpha-synuclein mutations exhibit defective axonal transport in cultured neurons. *J. Cell Sci.* 117 1017–1024. 10.1242/jcs.00967 14996933

[B65] Sekigawa A. Takamatsu Y. Sekiyama K. Hashimoto M. (2015). Role of alpha- and beta-synucleins in the axonal pathology of parkinson’s disease and related synucleinopathies. *Biomolecules* 5 1000–1011. 10.3390/biom5021000 25996784 PMC4496706

[B66] Shahmoradian S. H. Genoud C. Graff-Meyer A. Hench J. Moors T. Schweighauser G. (2017a). Lewy pathology in Parkinson’s disease consists of a crowded organellar membranous medley. *bioRxiv* [Preprint]. 137976 10.1101/137976 31235907

[B67] Shahmoradian S. H. Tsai E. H. R. Diaz A. Guizar-Sicairos M. Raabe J. Spycher L. (2017b). Three-dimensional imaging of biological tissue by cryo X-ray ptychography. *Sci. Rep.* 7:6291. 10.1038/s41598-017-05587-4 28740127 PMC5524705

[B68] Shu X. Lev-Ram V. Deerinck T. J. Qi Y. Ramko E. B. Davidson M. W. (2011). A genetically encoded tag for correlated light and electron microscopy of intact cells, tissues, and organisms. *PLoS Biol.* 9:e1001041. 10.1371/journal.pbio.1001041 21483721 PMC3071375

[B69] Simons M. Nave K. A. (2015). Oligodendrocytes: myelination and axonal support. *Cold Spring Harb. Perspect. Biol.* 8:a020479. 10.1101/cshperspect.a020479 26101081 PMC4691794

[B70] Su J. H. Cummings B. J. Cotman C. W. (1993). Identification and distribution of axonal dystrophic neurites in Alzheimer’s disease. *Brain Res.* 625 228–237. 10.1016/0006-8993(93)91063-X 8275305

[B71] Tagliaferro P. Burke R. E. (2016). Retrograde axonal degeneration in parkinson disease. *J. Parkinsons Dis.* 6 1–15. 10.3233/JPD-150769 27003783 PMC4927911

[B72] Thal D. R. Capetillo-Zarate E. Del Tredici K. Braak H. (2006). The development of amyloid beta protein deposits in the aged brain. *Sci. Aging Knowledge Environ.* 2006:re1. 10.1126/sageke.2006.6.re1 16525193

[B73] Thibault P. Dierolf M. Menzel A. Bunk O. David C. Pfeiffer F. (2008). High-resolution scanning x-ray diffraction microscopy. *Science* 321 379–382. 10.1126/science.1158573 18635796

[B74] Thibault P. Guizar-Sicairos M. (2012). Maximum-likelihood refinement for coherent diffractive imaging. *New J. Physics* 14:063004 10.1088/1367-2630/14/6/063004

[B75] Todorich B. Pasquini J. M. Garcia C. I. Paez P. M. Connor J. R. (2009). Oligodendrocytes and myelination: the role of iron. *Glia* 57 467–478. 10.1002/glia.20784 18837051

[B76] Tokuyasu K. (1986). Application of cryoultramicrotomy to immunocytochemistry. *J. Microscopy* 143 139–149. 10.1111/j.1365-2818.1986.tb02772.x 3531524

[B77] Tokuyasu K. T. (1980). Immunochemistry on ultrathin frozen sections. *Histochem. J.* 12 381–403. 10.1007/BF01011956 7440248

[B78] Uchihara T. (2007). Silver diagnosis in neuropathology: principles, practice and revised interpretation. *Acta Neuropathol.* 113 483–499. 10.1007/s00401-007-0200-2 17401570 PMC1868652

[B79] Van Heel M. Schatz M. (2005). Fourier shell correlation threshold criteria. *J. Struct. Biol.* 151 250–262. 10.1016/j.jsb.2005.05.009 16125414

[B80] Wakonig K. Stadler H. C. Odstrcil M. Tsai E. H. R. Diaz A. Holler M. (2020). PtychoShelves, a high-level, versatile framework for high-performance analysis of ptychographic data. *J. Appl. Crystallogr.* 10.1107/S1600576720001776 32280327 PMC7133065

[B81] Ward R. J. Zucca F. A. Duyn J. H. Crichton R. R. Zecca L. (2014). The role of iron in brain ageing and neurodegenerative disorders. *Lancet Neurol.* 13 1045–1060. 10.1016/S1474-4422(14)70117-6 25231526 PMC5672917

[B82] Wu H. R. Chen S. T. Chu Y. S. Conley R. Bouet N. Chien C. C. (2012). Nanoresolution radiology of neurons. *J. Phys. D Appl. Phys.* 45:242001 10.1088/0022-3727/45/24/242001

[B83] Zucca F. A. Vanna R. Cupaioli F. A. Bellei C. De Palma A. Di Silvestre D. (2018). Neuromelanin organelles are specialized autolysosomes that accumulate undegraded proteins and lipids in aging human brain and are likely involved in Parkinson’s disease. *NPJ Parkinsons Dis.* 4:17. 10.1038/s41531-018-0050-8 PMC5988730 29900402

