# Supplementary material for: Alterations in Sub-Axonal Architecture Between Normal Aging and Parkinson’s Diseased Human Brains Using Label-Free Cryogenic X-ray Nanotomography
Source: Front Neurosci. 2020 Nov 25;14:570019. doi: 10.3389/fnins.2020.570019 (PMC7724048; doi:10.3389/fnins.2020.570019)
Supplement: Supplementary file 1 [file Data_Sheet_1.DOCX]

Supplementary Material


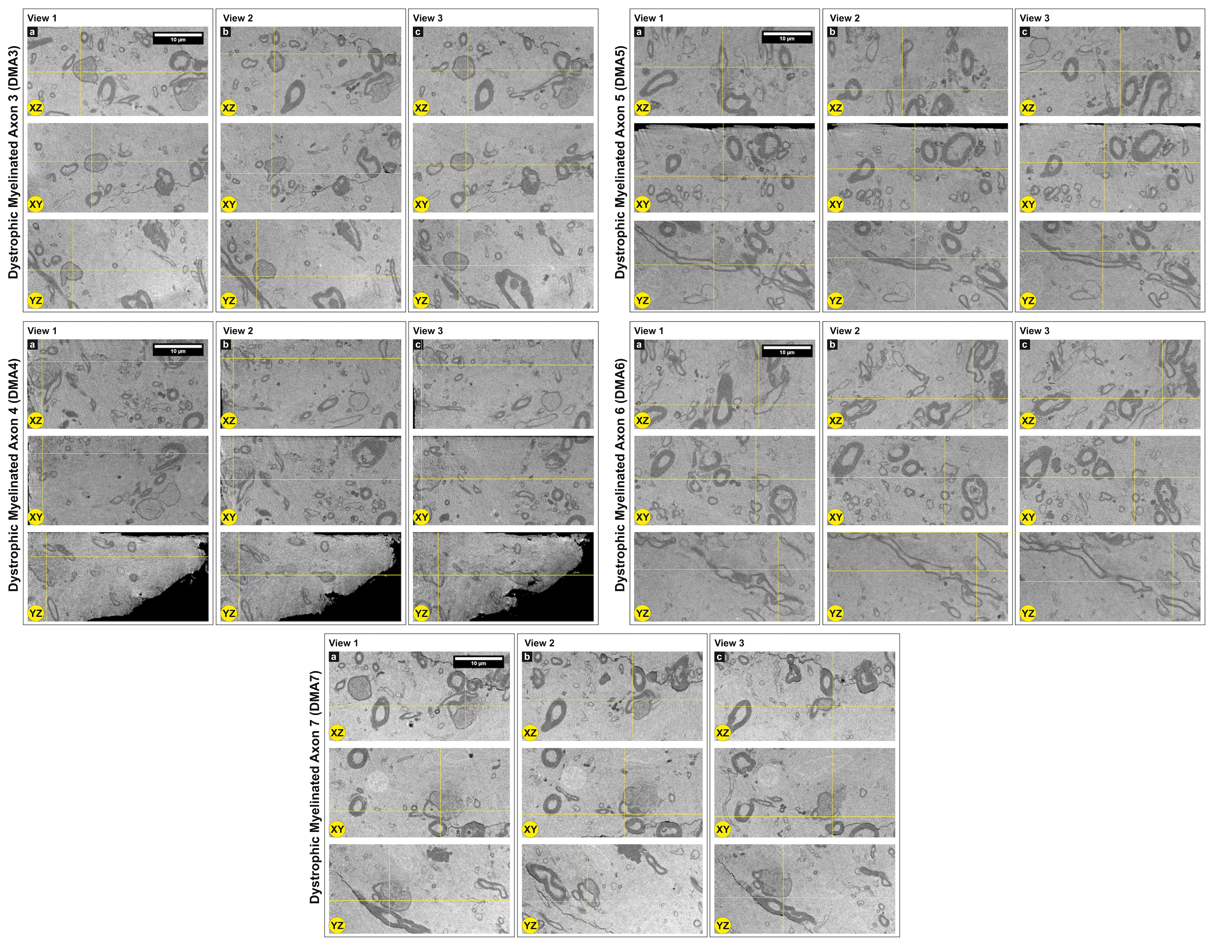


**Supplementary Figure 1. Dystrophic myelinated axons (DMAs) by cryo-PXCT in Parkinson’s diseased human brain.** Single 2D orthoslices with inverted grayscale showing the appearance of five DMAs (indicated by the yellow crosses) in different planes (X-Z, X-Y, Y-Z) and different positions (Views 1, 2, 3) in the 3D tomogram. Yellow crosses indicate the position of the DMA in the different views and planes. Other DMAs are shown in Figures 4 and S2. Grayscale shown herein does not correspond directly to mass density as opposed to Figure 1. Scale bars: 10 µm.


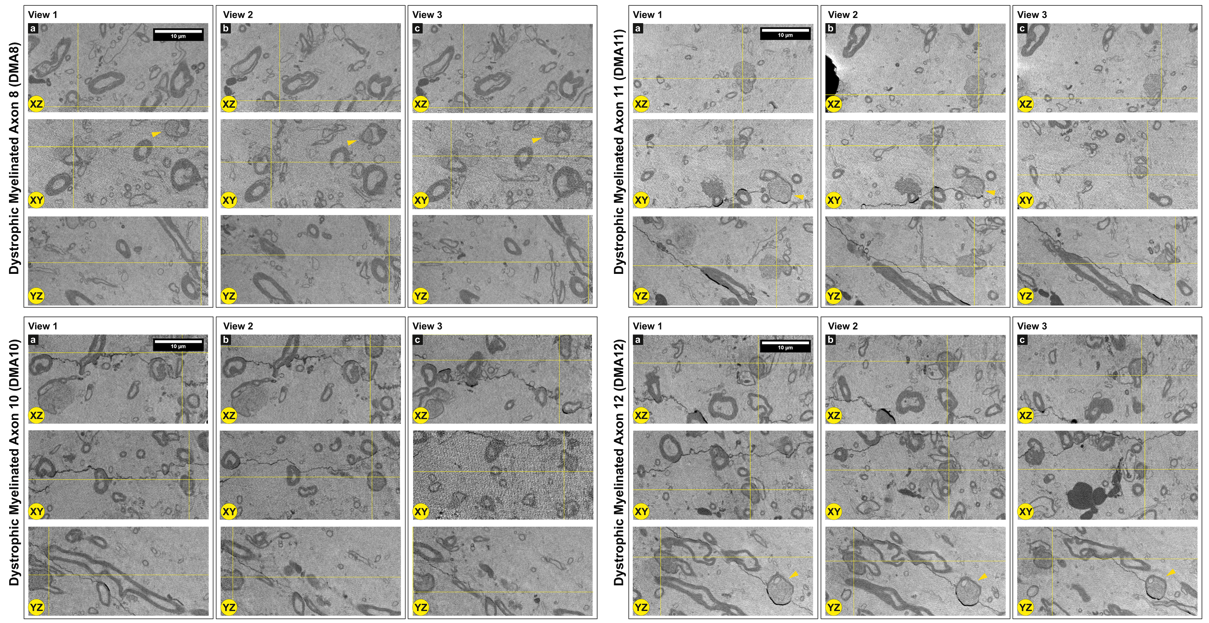


**Supplementary Figure 2. Dystrophic myelinated axons (DMAs) by cryo-PXCT in Parkinson’s diseased human brain.** Single 2D orthoslices with inverted grayscale showing the appearance of four DMAs (indicated by the yellow crosses) in different planes (X-Z, X-Y, Y-Z) and different positions (Views 1, 2, 3) in the 3D tomogram. Yellow crosses indicate the position of the DMA in the different views and planes. Yellow arrowheads indicate other DMAs present in the same viewing frame. Other DMAs are shown in Figures 4 and S1. Grayscale shown herein does not correspond directly to mass density as opposed to Figure 1. Scale bars: 10 µm.


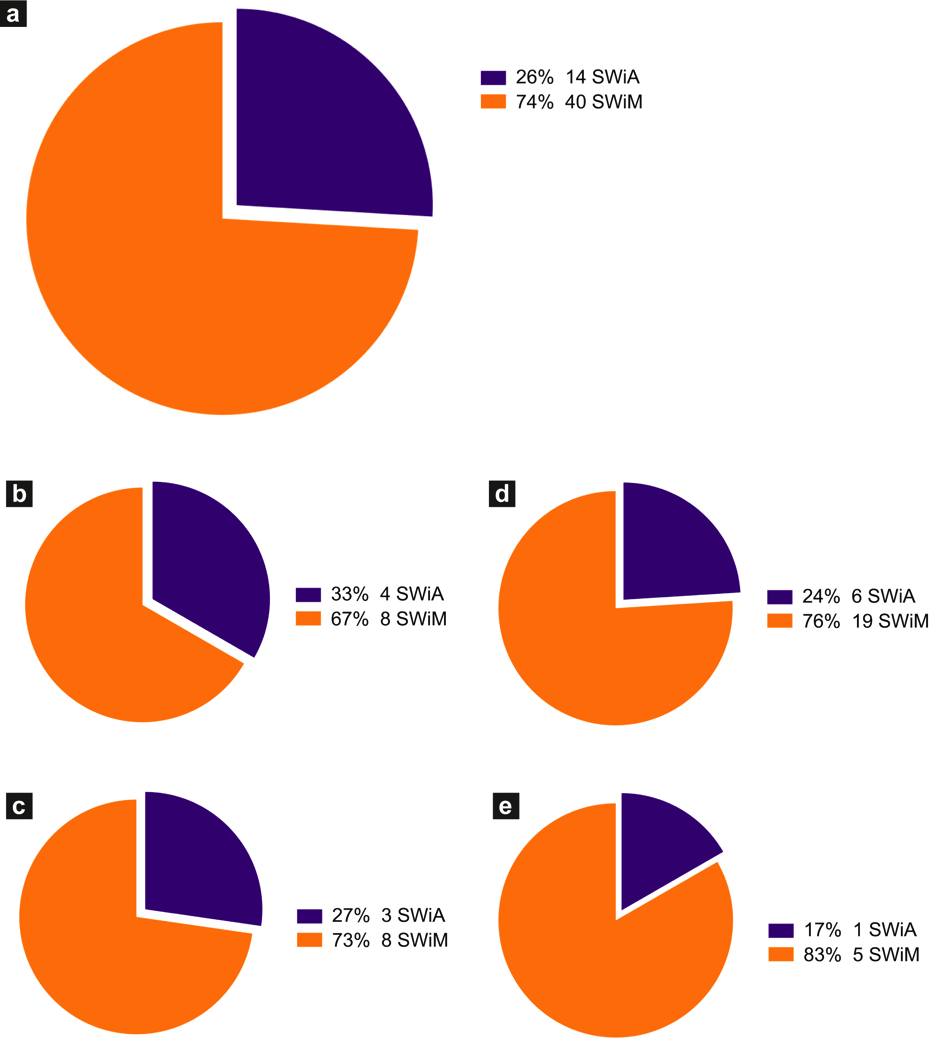


**Supplementary Figure 4. Type and abundance of DMA observed in cryo-PXCT tomograms of Parkinson’s diseased human brain.** DMAs were individually assessed and categorized as either SWiA type (swelling of neuronal cytoplasm) or SWiM type (swelling of oligodendrocyte cytoplasm within the myelin). (a) Of all four cryo-PXCT tomograms of PD human brain, 54 DMAs were identified, 40 (74%) corresponding to SWiM type, and 14 (26%) corresponding to SWiA type. Measurements for each individual tomogram correspond to (b) Tomo 3, (c) Tomo 4, (d) Tomo 1, and (e) Tomo 2, all of PD human brain. Characteristics of each matching tomogram are noted in Supplementary Table 2.


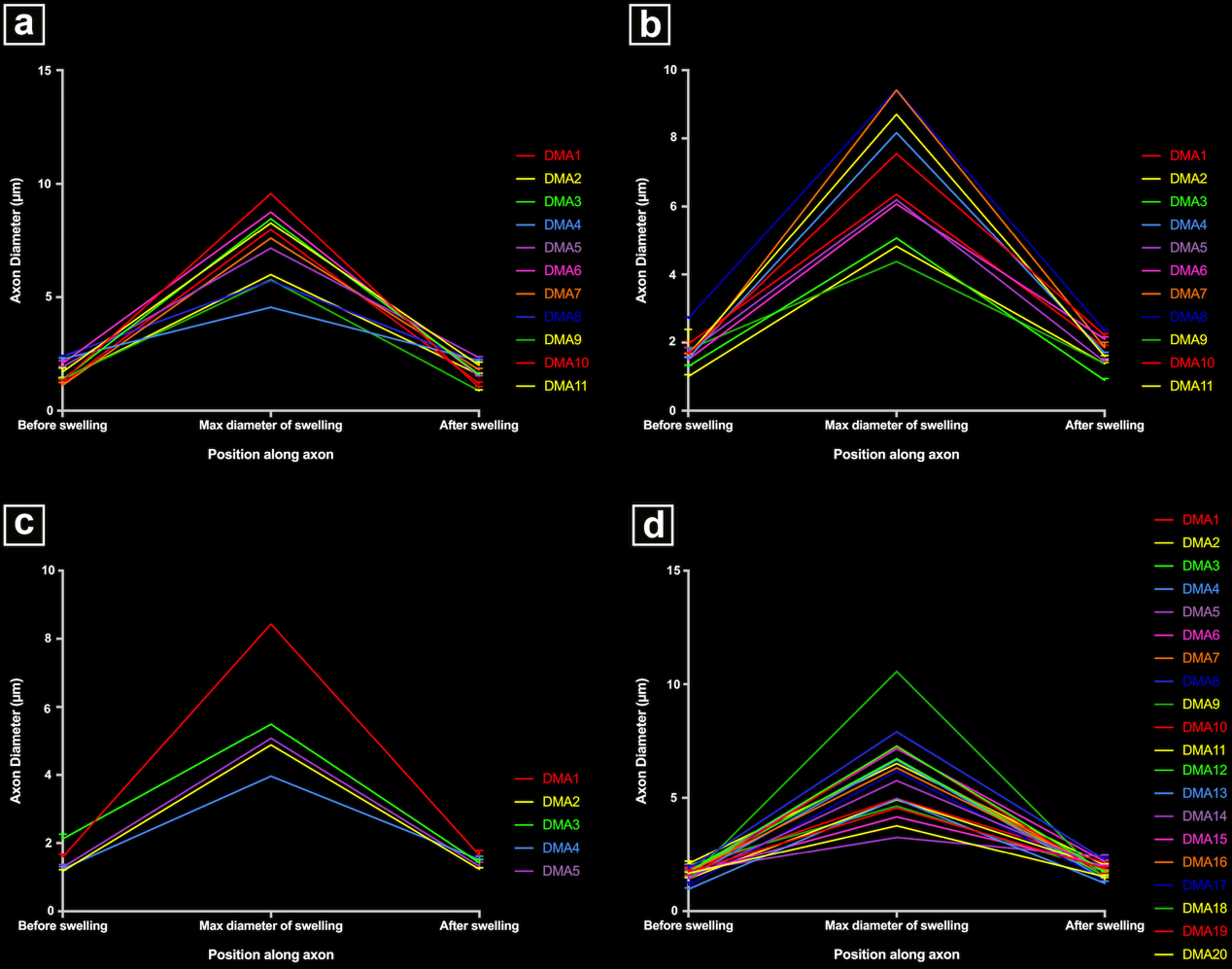


**Supplementary Figure 5. Measurements of DMAs in cryo-PXCT tomograms of Parkinson’s diseased human brain.** Measurements were collected at the widest point of the swelling of the DMA (indicated by “Max diameter of swelling” in the center point of the X-axis of each graph), and averaged across three positions along the axon immediately preceding the swelling (indicated by “Before swelling” on the X-axis of each graph), and averaged across three positions along the axon immediately following the swelling (indicated by “After swelling” on the X-axis of each graph). Measurements shown correspond to (a) Tomo 4, (b) Tomo 3, (c) Tomo 2, and (d) Tomo 1, all of PD human brain. Characteristics of each matching tomogram are noted in Supplementary Table 2.


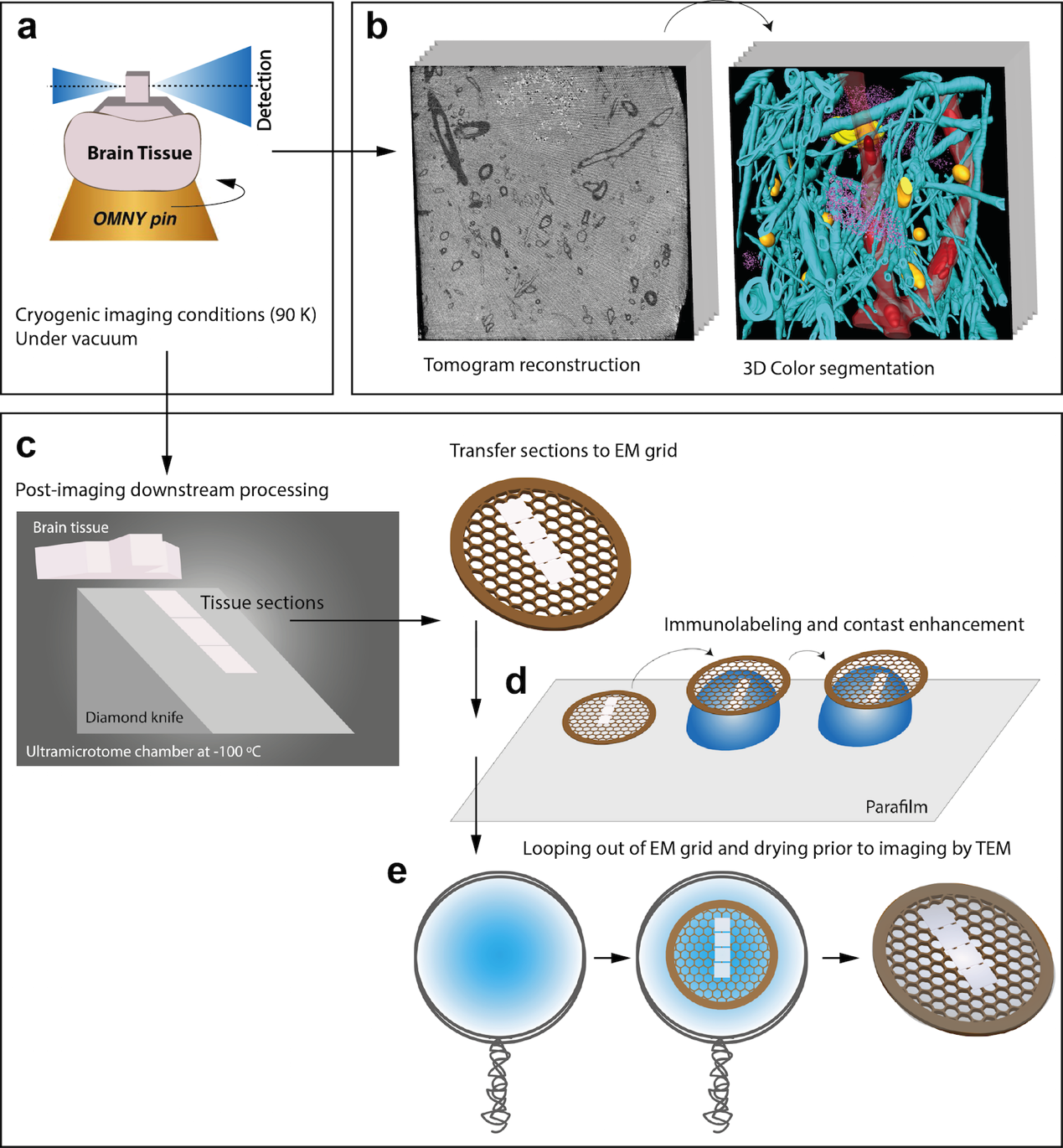


**Supplementary Figure 6. Simplified schematic workflow for tissue imaging by cryo-PXCT and downstream post-processing.** (a) Mounted and trimmed brain tissue is imaged under cryogenic imaging conditions (-180 ºC) under vacuum in the OMNY^21^ stage. The sample is sequentially rotated and imaged towards generating and reconstructing (b) a 3D volume, that can then be used for direct mass density measurements of features contained therein, and 3D color segmentation. Following cryo-PXCT, (c) the sample can then be re-mounted into the cryo-ultramicrotome chamber at -100 ºC for trimming as is established for cryo-immunogold electron microscopy^43^, then the resulting ultrathin sections cut by the diamond knife are transferred and thawed on an electron microscopy (EM) grid at ambient temperature, where they are subsequently placed section-side downwards onto droplets with the appropriate primary antibody, blocking buffer, secondary immunogold, etc., as previously described^44, 45^. (e) The sections on the EM grid are finally “looped out” with a mixture of methylcellulose and uranyl acetate (MC/UA) and left to dry, before removing the grid with the thin layer of MC/UA prior to imaging by transmission electron microscopy (TEM).
